# Viral co-detection of influenza virus and other respiratory viruses in hospitalized Brazilian patients during the first three years of the coronavirus disease (COVID)-19 pandemic: an epidemiological profile

**DOI:** 10.3389/fmicb.2024.1462802

**Published:** 2024-10-16

**Authors:** Bianca Aparecida Siqueira, Ketlyn Oliveira Bredariol, Matheus Negri Boschiero, Fernando Augusto Lima Marson

**Affiliations:** ^1^Laboratory of Molecular Biology and Genetics, São Francisco University, Bragança Paulista, Brazil; ^2^Laboratory of Clinical and Molecular Microbiology, São Francisco University, Bragança Paulista, Brazil; ^3^LunGuardian Research Group—Epidemiology of Respiratory and Infectious Diseases, São Francisco University, Bragança Paulista, Brazil; ^4^Medical Resident of Infectious Diseases at the Federal University of São Paulo, São Paulo, Brazil

**Keywords:** adenovirus, bocavirus, influenza virus, metapneumovirus, parainfluenza virus, respiratory syncytial virus, severe acute respiratory syndrome coronavirus 2, rhinovirus

## Abstract

**Introduction:**

In Brazil, few studies were performed regarding the co-detection of respiratory viruses in hospitalized patients. In this way, the study aimed to describe the epidemiological profile of hospitalized patients due to influenza virus infection that presented co-detection with another respiratory virus.

**Methods:**

The epidemiological analysis was made by collecting data from Open-Data-SUS. The study comprised patients infected by the influenza A or B virus with positive co-detection of another respiratory virus, such as adenovirus, bocavirus, metapneumovirus, parainfluenza virus (types 1, 2, 3, and 4), rhinovirus, and respiratory syncytial virus (RSV). The markers [gender, age, clinical signs and symptoms, comorbidities, need for intensive care unit (ICU) treatment, and need for ventilatory support] were associated with the chance of death. The data was collected during the first three years of the coronavirus disease (COVID)-19 pandemic—from December 19, 2019, to April 06, 2023.

**Results:**

A total of 477 patients were included, among them, the influenza A virus was detected in 400 (83.9%) cases. The co-detection occurred, respectively, for RSV (53.0%), rhinovirus (14.0%), adenovirus (13.4%), parainfluenza virus type 1 (10.7%), parainfluenza virus type 3 (5.2%), metapneumovirus (3.8%), parainfluenza virus type 2 (3.6%), bocavirus (3.4%), and parainfluenza virus type 4 (1.5%). The co-detection rate was higher in the male sex (50.7%), age between 0–12 years of age (65.8%), and white individuals (61.8%). The most common clinical symptoms were cough (90.6%), dyspnea (78.8%), and fever (78.6%). A total of 167 (35.0%) people had at least one comorbidity, mainly cardiopathy (14.3%), asthma (8.4%), and diabetes mellitus (7.3%). The need for ICU treatment occurred in 147 (30.8%) cases, with most of them needing ventilatory support (66.8%), mainly non-invasive ones (57.2%). A total of 33 (6.9%) patients died and the main predictors of death were bocavirus infection (OR = 14.78 [95%CI = 2.84–76.98]), metapneumovirus infection (OR = 8.50 [95%CI = 1.86–38.78]), race (other races vs. white people) (OR = 3.67 [95%CI = 1.39–9.74]), cardiopathy (OR = 3.48 [95%CI = 1.13–10.71]), and need for ICU treatment (OR = 7.64 [95%CI = 2.44–23.92]).

**Conclusion:**

Co-detection between the influenza virus and other respiratory viruses occurred, mainly with RSV, rhinovirus, and adenovirus being more common in men, white people, and in the juvenile phase. Co-detection of influenza virus with bocavirus and metapneumovirus was associated with an increased chance of death. Other factors such as race, cardiopathy, and the need for an ICU were also associated with a higher chance of death.

## Graphical abstract

^a^Other races included the patients self-declared as Black people, Asian individuals, Mixed individuals (*Pardos*), and Indigenous peoples. The data are presented using a *log-rank* scale. 95%CI, 95% confidence interval; N, number of individuals (cases)

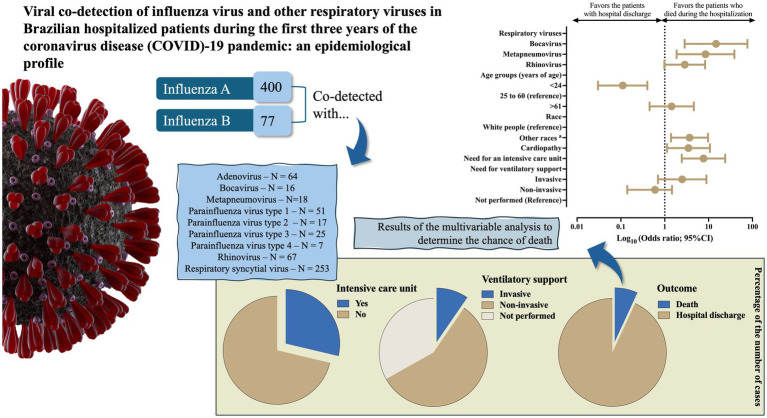

## 1 Introduction

The flu seasonality is marked by a high incidence of acute respiratory infection, which is frequently associated with enhanced hospitalizations due to viral infections. The main virus responsible for respiratory infection is the influenza virus. Hospitalizations resulting from infection with this virus represent an ongoing challenge for healthcare professionals, due to its ability to develop into more serious complications and even death, especially in high-risk groups (Iuliano et al., 2018; Chu et al., 2020; Lafond et al., 2021).

According to the World Health Organization, the incidence of influenza worldwide can be up to 1 billion cases, being the most common respiratory infection (The burden of Influenza, 2024). Nearly 3–5 million cases of influenza can develop into severe cases, with the need for hospitalizations, being that, among those hospitalized with influenza, severe cases can vary between 5–24% (Ao et al., 2019). Most of the patients who needed an intensive care unit (ICU) or even those who died were children, had comorbidities, were also diagnosed with pneumonia, lived in large households, and prior seeking care before hospitalization (Ao et al., 2019). Furthermore, influenza infection has a relatively high mortality, with nearly 290–650 thousand deaths annually (The burden of Influenza, 2024).

The co-detection of the influenza virus and other viral agents is frequent and has significant implications for the epidemiology and treatment of the patient (Trenholme et al., 2017; Choe et al., 2020). The precise identification of these additional viruses can be fundamental for the understanding of the disease severity and to evaluate the clinical outcomes and enhanced rate of hospitalization, by providing valuable information for infection prevention and control (Pinky and Dobrovolny, 2016; Szymański et al., 2017). Furthermore, a recent meta-analysis suggests that the co-infection of the influenza virus and other respiratory viruses, mainly the severe acute respiratory syndrome virus (SARS-CoV-2), can be associated with more severe and worse outcomes (Yan et al., 2023).

In Brazil, respiratory co-infections have a high prevalence despite the difficulty in identifying the etiological agent (Boschiero et al., 2022a). Frequently, the symptoms are treated without even considering proper underlying viral etiology, resulting in underreporting and underestimation of the true impact of viral infections (Alexander and Dobrovolny, 2022). The diagnostic gap can affect the efficacy of control and prevention measures, highlighting the urgency of a comprehensive approach in the evaluation of respiratory infections, especially those associated with influenza viruses and other viral agents (Gregianini et al., 2019; Martin-Loeches et al., 2019).

Thus, this study aims to analyze the prevalence of co-detection between influenza virus and other respiratory viruses, except SARS-CoV-2, in Brazilian hospitalized patients during the coronavirus disease (COVID)-19 pandemic.

## 2 Methods

### 2.1 General aspects and epidemiology analysis

An epidemiological analysis was conducted using data available at Open-Data-SUS.^1^ All the data have been imputed according to the severe acute respiratory infection surveillance [Information System Platform for Influenza Epidemiological Surveillance—from Portuguese *Sistema de Informação da Vigilância Epidemiológica da Gripe* (SIVEP-Gripe)] administered by the Brazilian Ministry of Health, which gained relevance due to the swine flu pandemic (H1N1) (de Souza et al., 2020). The data was collected from December 19, 2019, to April 6, 2023—three years since the beginning of the COVID-19 pandemic in Brazil. The dataset described in the article has been used for several other similar studies, especially, during the COVID-19 pandemic, being considered robust data for studies of epidemiology aiming to increase the quality of public healthcare policies in Brazil (Baqui et al., 2020; Boschiero et al., 2022b; Sansone et al., 2022b).

A descriptive analysis of the epidemiological profile of hospitalized patients with severe acute respiratory syndrome due to influenza virus infection and the co-detection of other respiratory viruses was performed. It was also performed an association between epidemiological profile and the chance of death. The viral profile was performed according to molecular (real-time polymerase chain reaction) tests. In some cases, biochemical immunological tests (antibody and antigen tests) were also performed depending on the availability of tests in the institution that imputed the data into the surveillance system. The Brazilian Ministry of Health defined a severe case as any patient who presented dyspnea OR peripheral oxygen saturation <95% OR any sign of respiratory distress (for example cyanosis and the use of accessory muscle) (Ministério da Saúde, 2024).

**Inclusion criteria**: all individuals who have been hospitalized during the COVID-19 pandemic due to influenza A or B virus infection with co-detection for other viruses [adenovirus, bocavirus, metapneumovirus, parainfluenza virus (types 1, 2, 3, and 4), rhinovirus, and respiratory syncytial virus (RSV)] were included.

**Exclusion criteria**: individuals who presented a positive SARS-CoV-2 test have been excluded from the analysis. Concomitantly, the following individuals were not included: (i) individuals without data on the main outcome—death or hospital discharge; (ii) individuals without any laboratory test that confirms viral infection—molecular test; (iii) foreigners hospitalized in Brazil; (iv) individuals infected with influenza, but not specified if it is influenza A or B; and (v) individuals who died during hospitalization due to other causes not described in the dataset.

### 2.2 Data acquisition

Initially, the data were acquired as a .csv file from Open-Data-SUS (see text footnote 1). The structure of the file was analyzed using the Statistical Package for the Social Sciences (SPSS) software (IBM SPSS Statistics for Macintosh, Version 27.0, IBM inc. Armonk NY, United States). After the first visualization of the dataset, the raw data was acquired and analyzed. The following markers were described:

(i) Demographic profile, including federal unity of the residence (states and federal district), date of the hospitalization due to severe acute respiratory syndrome, gender (female and male), age (<1 year of age, 1–12 years of age, 13–24 years of age, 25–60 years of age, 61–72 years of age, 73–85 years of age, and +85 years of age) (Dyussenbayev, 2017), self-declared race [White people, Black people, multiracial background (in Brazil described as Mixed or *Pardos* individuals), Asian individuals, and Indigenous peoples] (Sze et al., 2020; Sansone et al., 2022b; Corouraça|Educa|Jovens—IBGE, 2024), educational level (no education/illiterate, primary education of 1° cycle, primary education of 2° cycle, high school, and college), and place of residence (urban or rural and peri-urban).

(ii) Data regarding the type and subtype of the virus infection, such as influenza A and B, adenovirus, bocavirus, metapneumovirus, parainfluenza virus (types 1, 2, 3, and 4), rhinovirus and RSV, residence in a region of flu outbreak, nosocomial infection, Flu vaccination status, and use of antiviral drugs. The patients were grouped according to the number of co-detections described as follows: one co-detected virus, two co-detected viruses, and three co-detected viruses. Furthermore, the patients were distributed according to the date of notification, as well as the month of notification and the seasons (summer, autumn, spring, and winter).

(iii) Presence of comorbidities [comorbidity (any)—presence of at least one comorbidity, cardiopathy, hematological disorder, Down syndrome, hepatic disorder, asthma, diabetes mellitus, neurological disorder, chronic respiratory disorder, immunosuppression, kidney disorder, obesity, and other comorbidities (excluding the previous ones)].

(iv) Clinical signs and symptoms related to severe acute respiratory syndrome [fever, cough, sore throat, dyspnea, respiratory discomfort, peripheral oxygen saturation <95%, diarrhea, vomiting, abdominal pain, fatigue, loss of smell, loss of taste, and other clinical signs and symptoms (excluding the previous ones)].

(v) Results of image tests performed during viral infection. In the dataset, the results were imputed using two types of tests: x-ray of the thorax and high-resolution computed tomography of the thorax.

(vi) Need for ICU treatment, need for mechanical ventilatory support (not performed, non-invasive mechanical ventilatory support, and invasive mechanical ventilatory support), and outcomes (death or hospital discharge).

For better precision, two researchers (MB and FM) revised all the clinical and epidemiological data included in the study. The categorical data were numerically assigned to perform the missing data imputation and to carry out descriptive and inferential statistical analyses. The SPSS dataset was saved as an .xls file to perform the imputation of missing data values.

### 2.3 Missing data imputation

The inclusion of missing data for some features was performed because (i) the dataset had more than 5% missing data, (ii) the dataset did not have missing data only for the dependent variable, and (iii) the authors assumed that the variables were missing completely at random. Also, the characteristics that had more than 40% missing data were excluded. The missing data were imputed by the XLSTAT Statistical Software for Excel (Addinsoft Inc., Paris, Île-de-France, France) using the NIPALS (Nonlinear Iterative Partial Least Squares) algorithm. The XLSTAT Statistical Software generated a new Excel (.xls) data set used to perform the inferential statistical analyses in the SPSS software.

### 2.4 Statistical analysis

#### 2.4.1 Descriptive analysis

The descriptive analysis was conducted using the number of individuals (*N*) and the percentage (%) for categorical data. In the results of the inferential statistical analysis, when applicable, the Odds Ratio (OR) with its 95% confidence interval (95%CI) was also calculated.

#### 2.4.2 Bivariate analysis

A bivariate analysis was performed using the SPSS and OpenEpi (OpenEpi: Open-Source Epidemiological Statistics for Public Health, version. www.OpenEpi.com, April 04, 2013) softwares (Sullivan et al., 2009). The Chi-square test or Fisher’s exact test was used to estimate the distribution of the clinical and epidemiological markers with respect to the outcomes (death or hospital discharge). The OR with 95%CI was presented with its respective values. The OR was calculated using the OpenEpi software for 2 × 2 tables, all the values being manually included for each patient.

#### 2.4.3 Multivariable analysis

The multivariable analysis was done using the Binary Logistic Regression model with the Backward Stepwise method. Markers with *p* ≤ 0.05 in the bivariate analysis were included in the regression model. The dependent variable was the health outcome (death or recovery—hospital discharge). The data for (any) comorbidity (presence of at least one comorbidity) or others, symptoms (others), and characteristics of the patients with *p* > 0.05 were not used in this model. In the Logistic Regression model, the following information was presented: (i) coefficient B [including the SE (standard error)], which for the constant was called the intercept, (ii) the Wald Chi-square test and its significance, (iii) degrees of freedom for the Wald Chi-square test, and (iv) the Exp (*B*) which represents the exponentiation of the *B* coefficient (OR) including its 95%CI. The multicollinearity among the study markers was tested considering cut-off points <0.1 for tolerance and >10 for the variance inflation factor before carrying out the statistical inference analysis.

The results were compiled into tables and figures. Figures were created using GraphPad Prism version 10.2.3 for Mac (http://www.graphpad.com, GraphPad Software, San Diego CA, United States). The alpha error of 0.05 was considered in the bivariate and multivariable analyses carried out in the study.

### 2.5 Ethical aspects

The study was carried out in accordance with the Declaration of Helsinki and was approved by the institutional ethics committee (Certificate of Presentation for Ethical Appreciation No. 67241323.0.0000.5514; Study Approval No. 5.908.611).

## 3 Results

### 3.1 List of excluded markers

The markers that presented more than 40% of missing data were excluded, being the following markers excluded: educational level (*N* = 248, 52.0%), place of residence in a region of flu outbreak (*N* = 443, 92.9%), abdominal pain (*N* = 192, 40.3%), flu vaccination (*N* = 335, 70.2%), loss of smell (*N* = 191, 40.0%), loss of taste (*N* = 193, 40.3%), and image tests [thorax X-ray (*N* = 245, 51.4%) and thorax high resolution computed tomography (*N* = 429, 89.9%)].

### 3.2 Frequency of the main respiratory viruses detected with influenza virus

**Figure 1:**
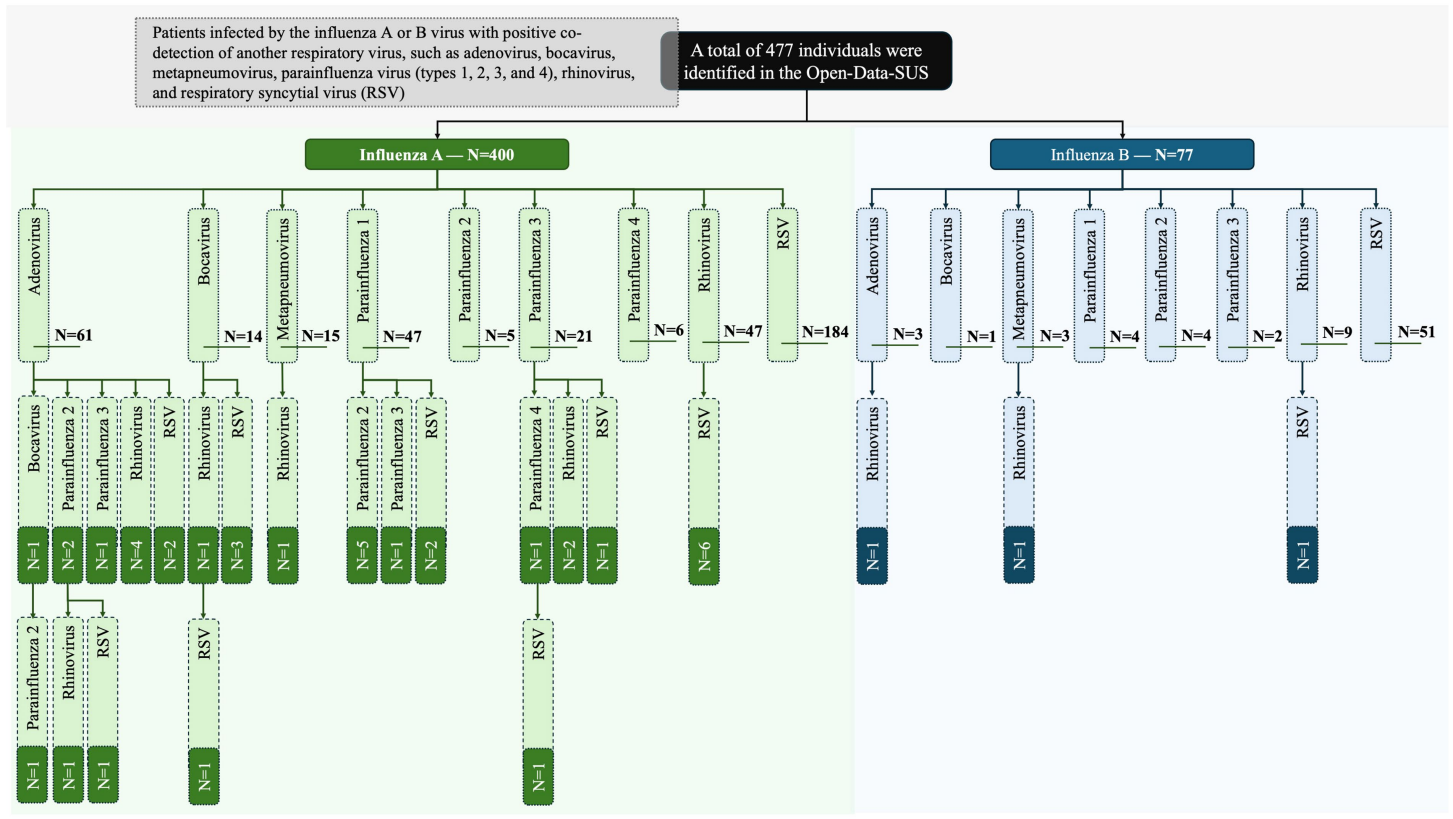
Epidemiological profile of the viral infection in Brazilian patients hospitalized for severe acute respiratory syndrome caused by the influenza virus. The data were collected in the Open-Data-SUS (https://opendatasus.saude.gov.br/). The data comprised the period from December 19, 2019, to April 06, 2023—three years since the beginning of the coronavirus disease (COVID)-19 pandemic in Brazil. *N*, number of individuals.

Influenza A was detected in 400 (83.9%) cases; in contrast, influenza B occurred only in 77 (16.1%) of the patients. Among the subtypes of influenza, when described in the dataset, the influenza A virus subtype H3N2 presented the highest co-detection rate with other viruses [183 (38.4%)] followed by the influenza A virus subtype H1N1 [50 (10.5%)]. Only one patient had the diagnosis of an influenza B subtype that was of a Victoria lineage. The co-detection occurred, respectively, for RSV [253 (53.0%)], rhinovirus [67 (14.0%)], adenovirus [64 (13.4%)], parainfluenza virus type 1 [51 (10.7%)], parainfluenza virus type 3 [25 (5.2%)], metapneumovirus [18 (3.8%)], parainfluenza virus type 2 [17 (3.6%)], bocavirus [16 (3.4%)], and parainfluenza virus type 4 [7 (1.5%)] (Table 1). A total of 31 (6.5%) individuals presented positive co-detection with two viruses; in addition, in 5 (1.0%) cases, the co-detection occurred for three viruses. The complete viral profile is presented as Figure 1 which includes all levels of co-detection. In addition, the Brazilian federal units with the highest rate of co-detection were São Paulo [156 (32.7%)], Paraná [66 (13.8%)], and Goiás [42 (8.8%)] states (Supplementary Table S1).

**Table 1:** Epidemiological profile of viral infection in Brazilian patients hospitalized for severe acute respiratory syndrome caused by the influenza virus.

Marker	Groups	*N* (%)
Influenza virus	Influenza A virus	167 (35.0%)
	Influenza A virus H1N1 subtype	50 (10.5%)
	Influenza A virus H3N2 subtype	183 (38.4%)
	Influenza B virus	76 (15.9%)
	Influenza B virus Victoria subtype	1 (0.2%)
Group of influenza virus	Influenza A virus	400 (83.9%)
	Influenza B virus	77 (16.1%)
Respiratory virus*	Adenovirus	64 (13.4%)
	Bocavirus	16 (3.4%)
	Metapneumovirus	18 (3.8%)
	Parainfluenza virus type 1	51 (10.7%)
	Parainfluenza virus type 2	17 (3.6%)
	Parainfluenza virus type 3	25 (5.2%)
	Parainfluenza virus type 4	7 (1.5%)
	Rhinovirus	67 (14.0%)
	Respiratory syncytial virus	253 (53.0%)

%, percentage; *N*, number of individuals. *: 36 patients had more than two respiratory viruses - 31 (6.5%) with two additional viruses and 5 (1.0%) with three additional viruses. The data were collected in the Open-Data-SUS (https://opendatasus.saude.gov.br/). The data comprised the period from December 19, 2019, to April 06, 2023—three years since the beginning of the coronavirus disease (COVID)-19 pandemic in Brazil.

### 3.3 Distribution of respiratory viruses according to the date of notification and seasons

**Figure 2:**
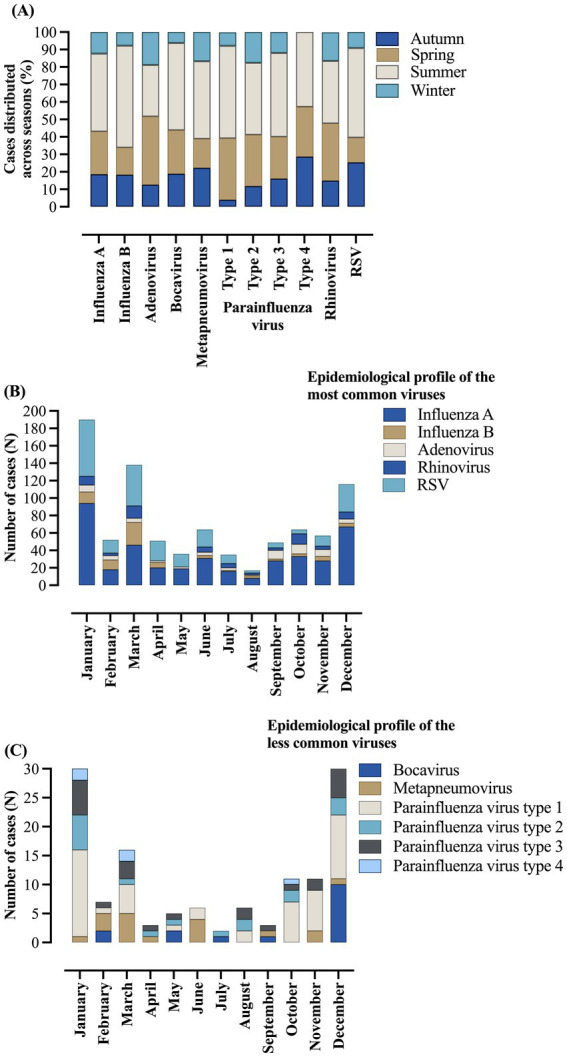
Distribution of respiratory viruses in hospitalized patients due to severe acute respiratory syndrome caused by influenza virus infection according to seasons and date of notification. **(A)** Number of cases distributed across seasons. **(B)** Epidemiological profile of the most common viruses according to date. **(C)** Epidemiological profile of the less common viruses according to date. The data comprised the period from December 19, 2019, to April 06, 2023—three years since the beginning of the coronavirus disease (COVID)-19 pandemic in Brazil. %, percentage; *N*, number of individuals; RSV, respiratory syncytial virus. Autumn from March 21 to June 21. Winter from June 21 to September 23. Spring from September 23 to December 21. Summer from December 21 to March 21. The dates were presented according to the notification periods.

In our data, the season with the highest number of cases was the summer [224 (47.0%)], followed by spring [110 (23.1%)], autumn [88 (18.4%)], and winter [55 (11.5%)] (Figure 2A; Supplementary Table S2). The viral profile was similar among all the viruses, except for some particularities such as adenovirus, which had the highest number of cases during the spring [25 (39.1%)]. The complete respiratory viral profile according to seasons is presented in Figure 2A and Supplementary Table S2. In addition, the distribution of the patients according to the date of notification and the month of notification is presented in Figures 2B,C and Supplementary Table S3.

### 3.4 Distribution of respiratory viruses according to age

**Figure 3:**
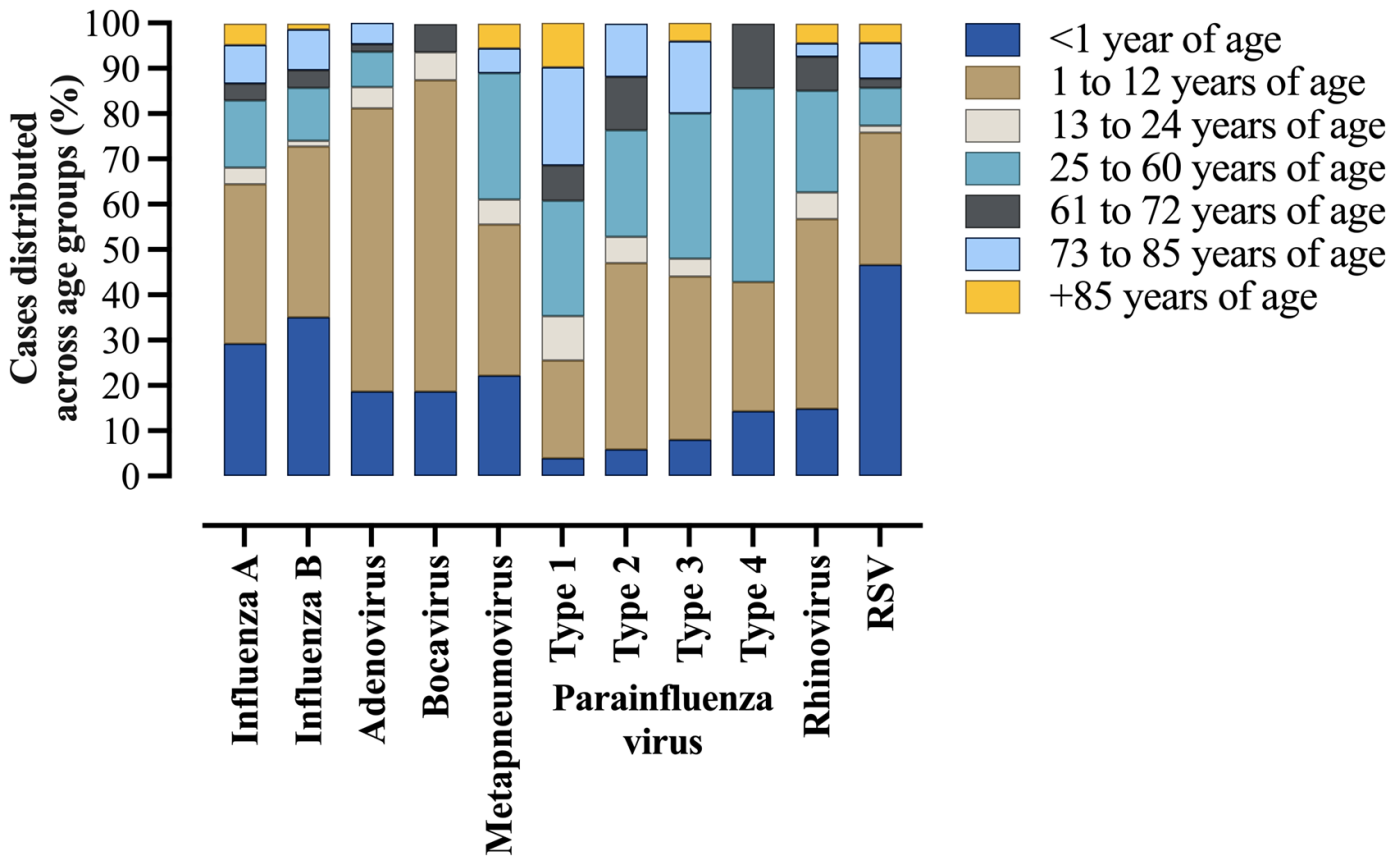
Distribution of respiratory viruses in hospitalized patients due to severe acute respiratory syndrome caused by influenza virus infection across age groups. The data comprised the period from December 19, 2019, to April 06, 2023—three years since the beginning of the coronavirus disease (COVID)-19 pandemic in Brazil. %, percentage; *N*, number of individuals; RSV, respiratory syncytial virus.

Most of the individuals with positive results for influenza A and B were <1 year of age (29.2 and 35%, respectively) and between 1 and 12 years of age (35.1 and 37.7%, respectively). In the same way, adenovirus (62.5%), bocavirus (68.7%), metapneumovirus (33.3%), parainfluenza virus type 2 (41.1%), parainfluenza virus type 3 (36%), and rhinovirus (41.8%) were more common in patients between 1 and 12 years of age. On the contrary, parainfluenza virus type 1 (25.5%) and parainfluenza virus type 4 (42.8%) were more common in patients between 25 and 60 years of age. Finally, RSV (46.6%) was more common in those <1 year of age. The complete data are presented in Figure 3 and Supplementary Table S4.

### 3.5 Clinical and epidemiological characteristics of the patients

**Figure 4:**
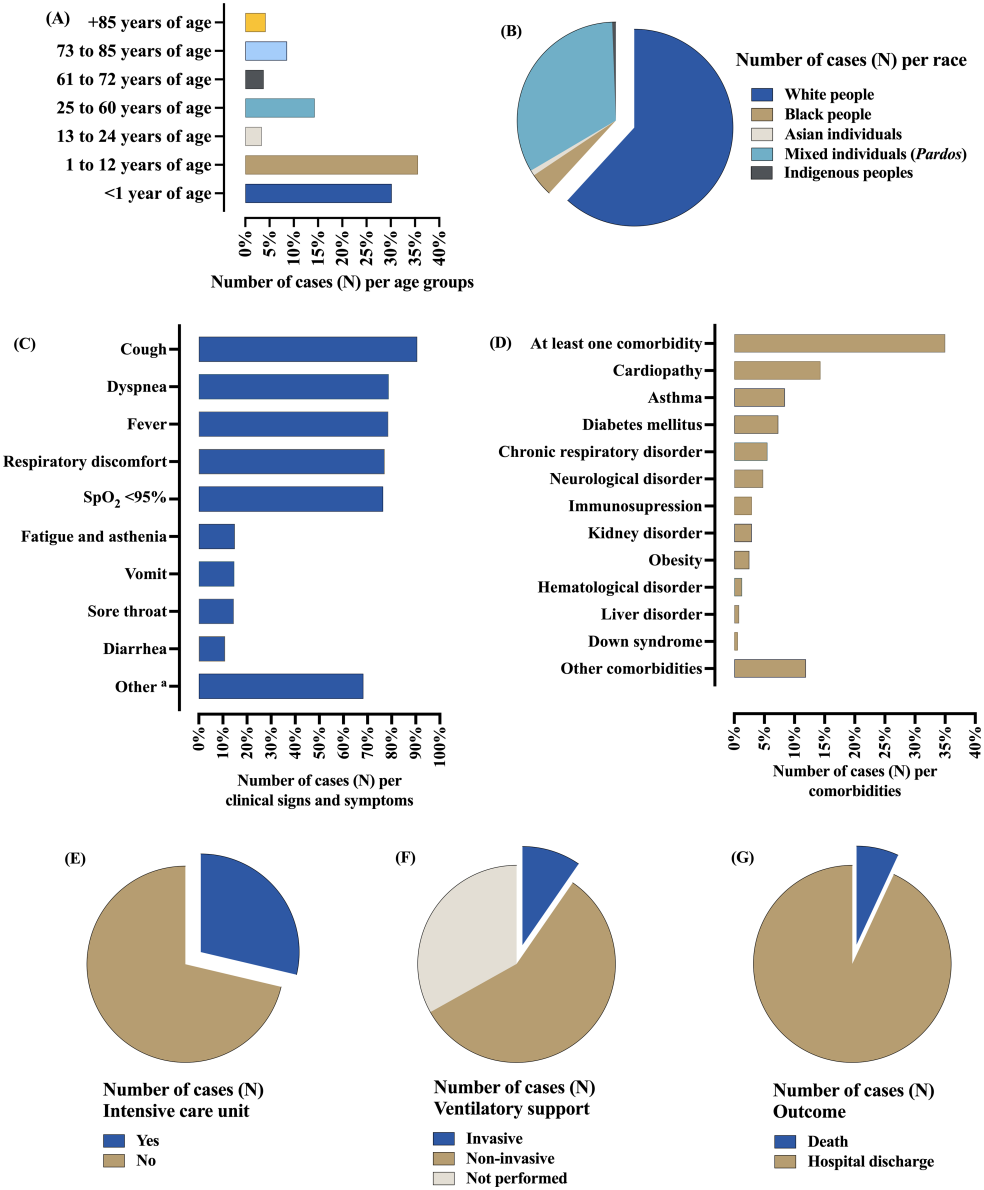
Epidemiological profile of Brazilian patients hospitalized for severe acute respiratory syndrome caused by the influenza virus who presented co-detection with other respiratory viruses. **(A)** Distribution of the patients according to the age groups. **(B)** Distribution of the patients according to the self-declared races. **(C)** Distribution of the patients according to clinical signs and symptoms. **(D)** Distribution of the patients according to the comorbidities. **(E)** Distribution of the patients according to the need for support from the intensive care. **(F)** Distribution of the patients according to the need for ventilatory support. **(G)** Distribution of the patients according to the outcome. The data comprised the period from December 19, 2019, to April 06, 2023—three years since the beginning of the coronavirus disease (COVID)-19 pandemic in Brazil. All the data is presented as the number of cases (N) in percentage. ^a^: Other clinical signs and symptoms summarize all the clinical signs and symptoms that were not listed previously in the dataset. %, percentage; *N*, number of individuals; SpO_2_, peripheral oxygen saturation.

In the hospitalized patients due to influenza infection, the co-detection rates of respiratory viruses were higher in the male sex [242 (50.7%)], age between zero and 12 years [314 (65.8%)], white people [295 (61.8%)], and the place of residence in urban areas [461 (96.6%)]. The nosocomial infection occurred only in 15 (3.1%) cases. The most common clinical signs and symptoms were cough [432 (90.6%)], dyspnea [376 (78.8%)], fever [375 (78.6%)], respiratory discomfort [368 (77.1%)], and peripheral oxygen saturation <95% [365 (76.5%)]. Regarding comorbidities, a total of 167 (35.0%) individuals presented at least one comorbidity, mainly cardiopathy [68 (14.3%)], asthma [40 (8.4%)], and diabetes mellitus [35 (7.3%)]. The use of antiviral drugs to treat flu symptoms was described in 137 (28.7%) cases. The need for ICU treatment occurred in 147 (30.8%) cases, with most of them needing ventilatory support [319 (66.8%)]—(i) non-invasive [273 (57.2%)] and (ii) invasive [46 (9.6%)]. Most of the patients had hospital discharge [444 (93.1%)], while 33 (6.9%) people died. The complete information is presented in Figure 4 and Table 2.

**Table 2:** Epidemiological profile of Brazilian patients hospitalized for severe acute respiratory syndrome caused by the influenza virus who presented co-detection with other respiratory viruses.

Marker	Group	*N* (%)
Gender	Male	242 (50.7%)
	Female	235 (49.3%)
Age	<1 year of age	144 (30.2%)
	1 to 12 years of age	170 (35.6%)
	13 to 24 years of age	16 (3.4%)
	25 to 60 years of age	68 (14.3%)
	61 to 72 years of age	18 (3.8%)
	73 to 85 years of age	41 (8.6%)
	+85 years of age	20 (4.2%)
Race	White people	295 (61.8%)
	Black people	18 (3.8%)
	Asian individuals	4 (0.8%)
	Mixed individuals (*Pardos*)	157 (32.9%)
	Indigenous peoples	3 (0.6%)
Place of residence	Urban	461 (96.6%)
	Rural + peri-urban	16 (3.4%)
Nosocomial infection	Yes	15 (3.1%)
	No	462 (96.9%)
Clinical signs and symptoms	Cough	432 (90.6%)
	Dyspnea	376 (78.8%)
	Fever	375 (78.6%)
	Respiratory discomfort	368 (77.1%)
	Peripheral oxygen saturation < 95%	365 (76.5%)
	Fatigue and asthenia	71 (14.9%)
	Vomit	70 (14.7%)
	Sore throat	69 (14.5%)
	Diarrhea	52 (10.9%)
	Other clinical signs and symptoms^a^	326 (68.3%)
Comorbidities	At least one comorbidity	167 (35.0%)
	Cardiopathy	68 (14.3%)
	Asthma	40 (8.4%)
	Diabetes mellitus	35 (7.3%)
	Chronic respiratory disorder	26 (5.5%)
	Neurological disorder	23 (4.8%)
	Immunosuppression	14 (2.9%)
	Kidney disorder	14 (2.9%)
	Obesity	12 (2.5%)
	Hematological disorder	6 (1.3%)
	Liver disorder	4 (0.8%)
	Down syndrome	3 (0.6%)
	Other comorbidities	57 (11.9%)
Use of antivirals to treat flu symptoms^b^	Yes	137 (28.7%)
	No	340 (71.3%)
Need for an intensive care unit	Yes	147 (30.8%)
	No	330 (69.2%)
Need for ventilatory support	Invasive	46 (9.6%)
	Non-invasive	273 (57.2%)
	Not performed	158 (33.1%)
Outcome	Hospital discharge	444 (93.1%)
	Death	33 (6.9%)

%, percentage; *N*, number of individuals.

a Other symptoms summarize all the symptoms that were not listed previously in the dataset.

b The antiviral therapy was used at the discretion of the attending physician; then it was not indicated based on the severe phenotype or the need for ventilatory support. The data were collected in the Open-Data-SUS (https://opendatasus.saude.gov.br/). The data comprised the period from December 19, 2019, to April 06, 2023—three years since the beginning of the coronavirus disease (COVID)-19 pandemic in Brazil.

### 3.6 Bivariate analysis to identify the death predictors in hospitalized patients

**Figure 5:**
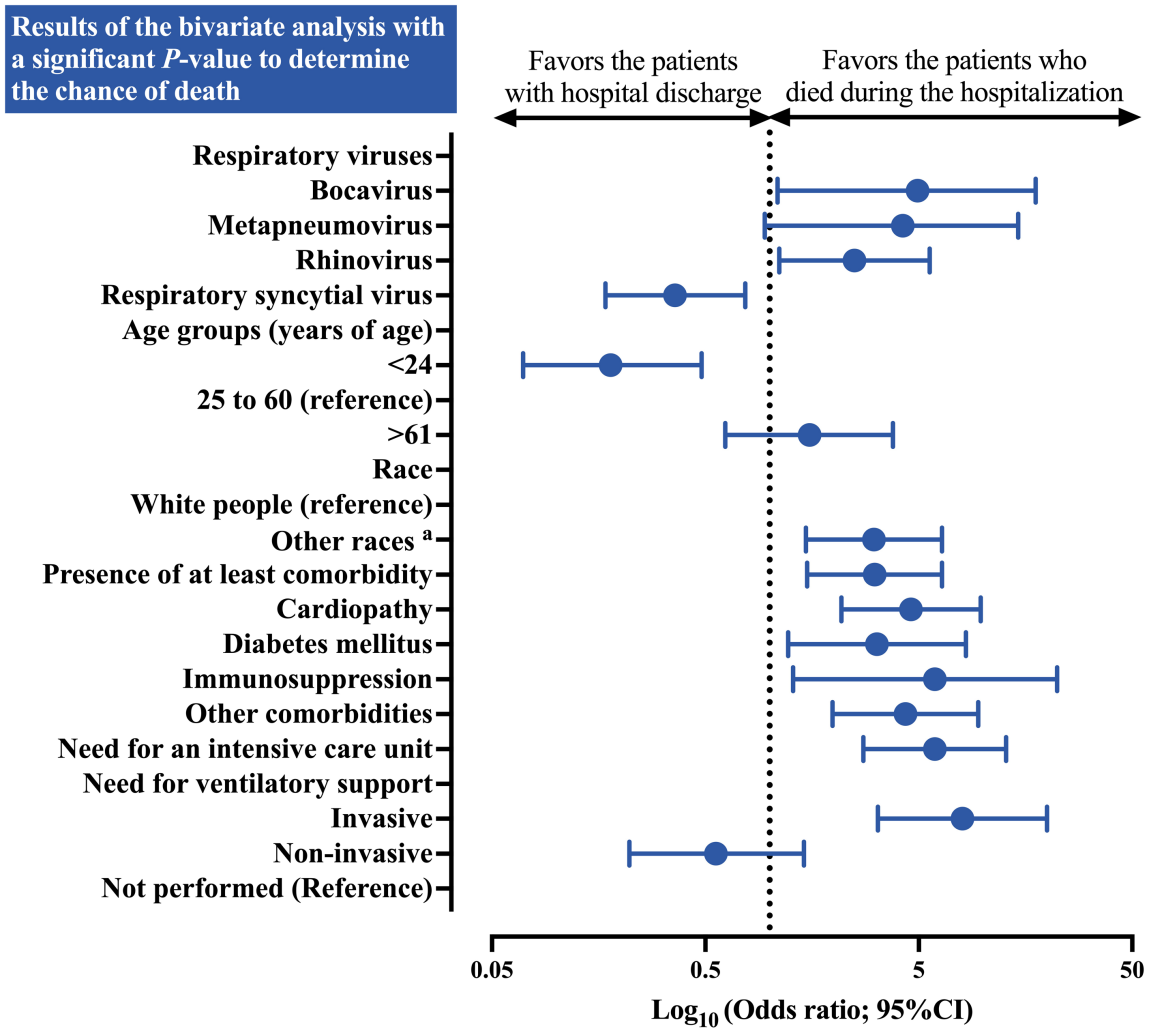
Association between the epidemiological profile of Brazilian patients hospitalized due to severe acute respiratory syndrome caused by the influenza virus who presented co-detection by other respiratory viruses and the outcomes after hospitalization. The figure presents the results of the bivariate analysis with a significant *p*-value exclusively to determine the probability of death. ^a^: Other races included the patients self-declared as Black people, Asian individuals, Mixed individuals (*Pardos*), and Indigenous peoples. The data comprised the period from December 19, 2019, to April 06, 2023—three years since the beginning of the coronavirus disease (COVID)-19 pandemic in Brazil. The Chi-square test or Fisher’s exact test was used to estimate the distribution of the clinical and epidemiological markers with respect to outcomes (death or hospital discharge). The alpha error of 0.05 was considered in the bivariate analyses carried out in the study. The data are presented using a *log-rank* scale. 95%CI, 95% confidence interval; OR, odds ratio.

A higher chance of death in hospitalized individuals with influenza was identified in the presence of co-detection with bocavirus (OR = 4.94 [95%CI = 1.09–17.66]) and rhinovirus (OR = 2.50 [95%CI = 1.11–5.63]). Furthermore, patients under 24 years of age presented a protective OR (OR = 0.18 [95%CI = 0.07–0.48]) for death compared to those between 25 and 60 years of age. Additionally, when all races were compared to the white race, an increased chance of death was described (OR = 3.08 [95%CI = 1.48–6.42]). Several other characteristics, such as the presence of at least one comorbidity (OR = 3.11 [95%CI = 1.50–6.42]), cardiopathy (OR = 4.60 [95%CI = 2.17–9.76]), diabetes mellitus (OR = 3.18 [95%CI = 1.22–8.32]), immunosuppression (OR = 5.94 [95%CI = 1.29–22.25]), and the presence of other comorbidities (OR = 4.33 [95%CI = 1.97–9.49]) were associated with a higher chance of death. The need for ICU (OR = 5.94 [95%CI = 2.75–12.83]) and invasive respiratory support (OR = 8.01 [95%CI = 3.22–19.95]) also increase the probability of death (Table 3; Figure 5).

**Table 3:** Association between the epidemiological profile of Brazilian patients hospitalized due to severe acute respiratory syndrome caused by the influenza virus who presented co-detection by other respiratory viruses and the outcomes after hospitalization.

Marker	Group	Death	Hospital discharge	Total	*p*-value	OR (95%CI)
Influenza A virus	Yes	28 (7.0%)	372 (93.0%)	400	1.000	1.08 (0.41–2.90)
	No	5 (6.5%)	72 (93.5%)	77		Reference
Influenza B virus	Yes	5 (6.5%)	72 (93.5%)	77	1.000	0.92 (0.34–2.47)
	No	28 (7.0%)	372 (93.0%)	400		Reference
Adenovirus	Yes	3 (4.7%)	61 (95.3%)	64	0.601	0.63 (0.12–2.12)
	No	30 (7.3%)	383 (92.7%)	413		Reference
Bocavirus	Yes	4 (25.0%)	12 (75.0%)	16	0.019	4.94 (1.09–17.66)
	No	29 (6.3%)	432 (93.7%)	461		Reference
Metapneumovirus	Yes	4 (22.2%)	14 (77.8%)	18	0.029	4.21 (0.95–14.60)
	No	29 (6.3%)	430 (93.7%)	459		Reference
Parainfluenza virus type 1	Yes	3 (5.9%)	48 (94.1%)	51	1.000	0.83 (0.16–2.81)
	No	30 (7.0%)	396 (93.0%)	426		Reference
Parainfluenza virus type 2	Yes	1 (5.9%)	16 (94.1%)	17	1.000	0.84 (0.02–5.73)
	No	32 (7.0%)	428 (93.0%)	460		Reference
Parainfluenza virus type 3	Yes	3 (12.0%)	22 (88.0%)	25	0.404	1.92 (0.35–6.94)
	No	30 (6.6%)	422 (93.4%)	452		Reference
Parainfluenza virus type 4	Yes	0 (0.0%)	7 (100.0%)	7	1.000	Not applicable
	No	33 (7.0%)	437 (93.0%)	470		-
Rhinovirus	Yes	9 (13.4%)	58 (86.6%)	67	0.035	2.50 (1.11–5.63)
	No	24 (5.9%)	386 (94.1%)	410		Reference
Respiratory syncytial virus	Yes	10 (4.0%)	243 (96.0%)	253	0.007	0.36 (0.17–0.77)
	No	23 (10.3%)	201 (89.7%)	224		Reference
Number of co-infections	One virus	30 (6.8%)	411 (93.2%)	441		Reference
	Two viruses	2 (6.5%)	29 (93.5%)	31	1.000	0.95 (0.10–4.05)
	Three viruses	1 (20.0%)	4 (80.0%)	5	0.607	3.41 (0.07–35.90)
Number of co-infections (grouped)	One virus	30 (6.8%)	411 (93.2%)	441		Reference
	+2 (two or three viruses)	3 (8.3%)	33 (91.7%)	36	0.925	1.25 (0.36–4.30)
Gender	Male	17 (7.0%)	225 (93.0%)	242	1.000	1.03 (0.51–2.10)
	Female	16 (6.8%)	219 (93.2%)	235		Reference
Age (years of age)	<24	9 (2.7%)	321 (97.3%)	330	<0.001	0.18 (0.07–0.48)
	25 to 60	9 (13.2%)	59 (86.8%)	68		Reference
	>61	15 (19.0%)	64 (81.0%)	79	0.473	1.54 (0.62–3.78)
Race	White people	12 (4.1%)	283 (95.9%)	295	<0.001	Reference
	Other races^a^	21 (11.5%)	161 (88.5%)	182		3.08 (1.48–6.42)
Place of residence	Urban	30 (6.5%)	431 (93.5%)	461	0.091	0.30 (0.08–1.12)
	Rural + peri-urban	3 (18.8%)	13 (81.2%)	16		Reference
Nosocomial infection	Yes	1 (6.7%)	14 (93.3%)	15	1.000	0.96 (0.02–6.71)
	No	32 (6.9%)	430 (93.1%)	462		Reference
Fever	Yes	23 (6.1%)	352 (93.9%)	375	0.269	0.60 (0.28–1.31)
	No	10 (9.8%)	92 (90.2%)	102		Reference
Cough	Yes	27 (6.2%)	405 (93.8%)	432	0.112	0.43 (0.17–1.11)
	No	6 (13.3%)	39 (86.7%)	45		Reference
Sore throat	Yes	6 (8.7%)	63 (91.3%)	69	0.606	1.34 (0.53–3.39)
	No	27 (6.6%)	381 (93.4%)	408		Reference
Dyspnea	Yes	30 (8.0%)	346 (92.0%)	376	0.119	2.83 (0.85–14.78)
	No	3 (3.0%)	98 (97.0%)	101		Reference
Respiratory discomfort	Yes	28 (7.6%)	340 (92.4%)	368	0.297	1.71 (0.65–4.55)
	No	5 (4.6%)	104 (95.4%)	109		Reference
Peripheral oxygen saturation	<95%	25 (6.8%)	340 (93.2%)	365	1.000	0.96 (0.42–2.18)
	≥95%	8 (7.1%)	104 (92.9%)	112		Reference
Diarrhea	Yes	3 (5.8%)	49 (94.2%)	52	1.000	0.81 (0.15–2.75)
	No	30 (7.1%)	395 (92.9%)	425		Reference
Vomit	Yes	1 (1.4%)	69 (98.6%)	70	0.069	0.17 (<0.01–1.06)
	No	32 (7.9%)	375 (92.1%)	407		Reference
Fatigue and asthenia	Yes	5 (7.0%)	66 (93.0%)	71	1.000	1.02 (0.38–2.74)
	No	28 (6.9%)	378 (93.1%)	406		Reference
Other clinical signs and symptoms^b^	Yes	21 (6.4%)	305 (93.6%)	326	0.563	0.80 (0.38–1.67)
	No	12 (7.9%)	139 (92.1%)	151		Reference
Presence of at least one comorbidity	Yes	20 (12.0%)	147 (88.0%)	167	0.002	3.11 (1.50–6.42)
	No	13 (4.2%)	297 (95.8%)	310		Reference
Cardiopathy	Yes	13 (19.1%)	55 (80.9%)	68	<0.001	4.60 (2.17–9.76)
	No	20 (4.9%)	389 (95.1%)	409		Reference
Hematological disorder	Yes	0 (0.0%)	6 (100.0%)	6	1.000	Not applicable
	No	33 (7.0%)	438 (93.0%)	471		-
Down syndrome	Yes	0 (0.0%)	3 (100.0%)	3	1.000	Not applicable
	No	33 (7.0%)	441 (93.0%)	474		-
Liver disorder	Yes	2 (50.0%)	2 (50.0%)	4	0.026	14.05 (0.99–199.9)
	No	31 (6.6%)	442 (93.4%)	473		Reference
Asthma	Yes	0 (0.0%)	40 (100.0%)	40	0.098	Not applicable
	No	33 (7.6%)	404 (92.4%)	437		-
Diabetes mellitus	Yes	6 (17.1%)	29 (82.9%)	35	0.026	3.18 (1.22–8.32)
	No	27 (6.1%)	415 (93.9%)	442		Reference
Neurological disorder	Yes	3 (13.0%)	20 (87.0%)	23	0.207	2.12 (0.38–7.76)
	No	30 (6.6%)	424 (93.4%)	454		Reference
Chronic respiratory disorder	Yes	4 (15.4%)	22 (84.6%)	26	0.096	2.64 (0.62–8.53)
	No	29 (6.4%)	422 (93.6%)	451		Reference
Immunosuppression	Yes	4 (28.6%)	10 (71.4%)	14	0.012	5.94 (1.29–22.25)
	No	29 (6.3%)	434 (93.7%)	463		Reference
Kidney disorder	Yes	3 (21.4%)	11 (78.6%)	14	0.065	3.92 (0.67–15.95)
	No	30 (6.5%)	433 (93.5%)	463		Reference
Obesity	Yes	1 (8.3%)	11 (91.7%)	12	0.581	1.23 (0.03–8.96)
	No	32 (6.9%)	433 (93.1%)	465		Reference
Other comorbidities	Yes	11 (19.3%)	46 (80.7%)	57	0.001	4.33 (1.97–9.49)
	No	22 (5.2%)	398 (94.8%)	420		Reference
Antivirals to treat the flu symptoms^c^	Yes	11 (8.0%)	126 (92.0%)	137	0.553	1.26 (0.59–2.68)
	No	22 (6.5%)	318 (93.5%)	340		Reference
Need for an intensive care unit	Yes	23 (15.6%)	124 (84.4%)	147	<0.001	5.94 (2.75–12.83)
	No	10 (3.0%)	320 (97.0%)	330		Reference
Need for ventilatory support	Invasive	15 (32.6%)	31 (67.4%)	46	<0.001	8.01 (3.22–19.95)
	Non-invasive	9 (3.3%)	264 (96.7%)	273	0.34	0.56 (0.22–1.45)
	Not performed	9 (5.7%)	149 (94.3%)	158		Reference

95%CI, 95% confidence interval; %, percentage; *N*, number of individuals; OR, odds ratio.

a Other races included the patients self-declared as Black people, Asian individuals, Mixed individuals (*Pardos*), and Indigenous peoples.

b Other clinical signs and symptoms summarize all the clinical signs and symptoms that were not listed previously in the dataset.

c The antiviral therapy was used at the discretion of the attending physician; then it was not indicated based on severe phenotype or the need for ventilatory support. The data were collected in the Open-Data-SUS (https://opendatasus.saude.gov.br/). The data comprised the period from December 19, 2019, to April 06, 2023—three years since the beginning of the coronavirus disease (COVID)-19 pandemic in Brazil. The Chi-square test or Fisher’s exact test was used to estimate the distribution of clinical and epidemiological markers with respect to outcomes (death or hospital discharge). The alpha error of 0.05 was considered in the bivariate analyses carried out in the study.

### 3.7 Multivariable analysis to identify the main predictors of death

**Figure 6:**
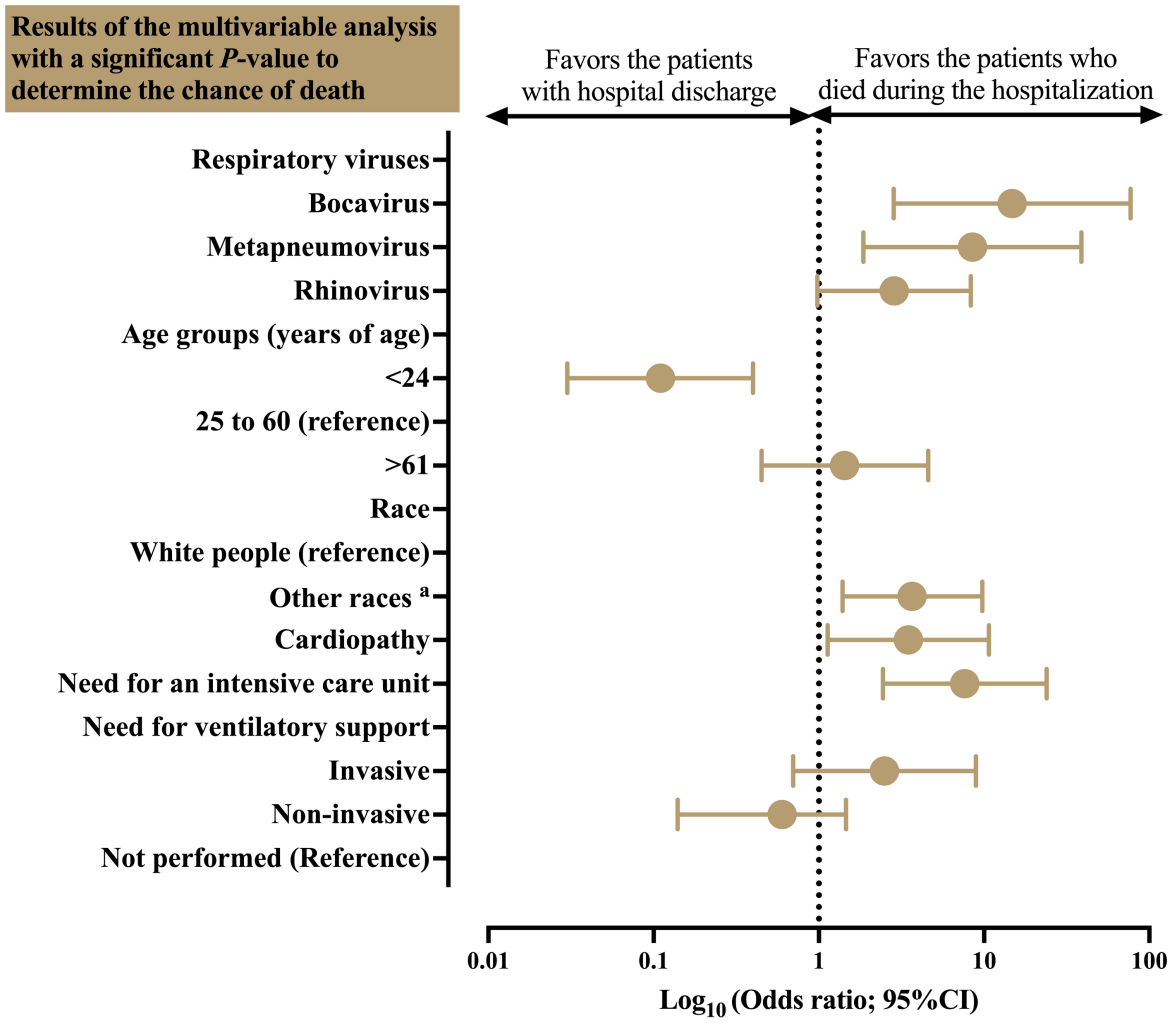
Multivariable analysis to identify the main predictors of death in Brazilian patients hospitalized due to severe acute respiratory syndrome caused by the influenza virus who presented co-detection by other respiratory viruses. The figure presents the results of the multivariable analysis (markers) with a significant *p*-value exclusively to determine the chance of death. ^a^: Other races included the patients self-declared as Black people, Asian individuals, Mixed individuals (*Pardos*), and Indigenous peoples. The following markers were included in the model: viral infection (bocavirus, metapneumovirus, rhinovirus, and respiratory syncytial virus), age, race, comorbidities (cardiopathy, hepatic disorder, diabetes mellitus, and immunodepression), need for an intensive care unit, and need for mechanical ventilatory support. The data comprised the period from December 19, 2019, to April 06, 2023—three years since the beginning of the coronavirus disease (COVID)-19 pandemic in Brazil. The multivariable analysis was done using the Binary Logistic Regression model with the Backward Stepwise method. Markers with *p* ≤ 0.05 in the bivariate analysis were included in the regression model. The dependent variable was the health outcome (death or recovery—hospital discharge). The alpha error of 0.05 was considered in the multivariable analysis carried out in the study. The data are presented using a *log-rank* scale. 95%CI, 95% confidence interval; OR, odds ratio.

The multivariable analysis to identify the main predictors of death in Brazilian patients hospitalized due to influenza virus infection who presented co-detection by other respiratory viruses highlights the importance of the following markers: bocavirus infection (OR = 14.78 [95%CI = 2.84–76.98]), metapneumovirus infection (OR = 8.50 [95%CI = 1.86–38.78]), race (other races versus white people) (OR = 3.67 [95%CI = 1.39–9.74]), presence of cardiopathy (OR = 3.48 [95%CI = 1.13–10.71]), and need for ICU (OR = 7.64 [95%CI = 2.44–23.92]) (Table 4; Figure 6).

**Table 4:** Multivariable analysis to identify the main predictors of death in Brazilian patients hospitalized due to severe acute respiratory syndrome caused by the influenza virus who presented co-detection by other respiratory viruses.

Markers	*B*	SE	Wald	df	*p*-value	OR	95%CI
Respiratory virus							
Bocavirus	2.69	0.84	10.23	1	0.001	14.78	2.84–76.98
Metapneumovirus	2.14	0.78	7.63	1	0.006	8.50	1.86–38.78
Rhinovirus	1.05	0.54	3.73	1	0.054	2.86	0.98–8.32
Age (years of age)							
<1 to 24	−2.26	0.68	11.10	1	0.001	0.11	0.03–0.40
25 to 60 (reference)			16.28	2	0.001		
>61	0.36	0.59	0.37	1	0.545	1.43	0.45–4.59
Race (Other vs. white people)^a^	1.30	0.50	6.84	1	0.009	3.67	1.39–9.74
Cardiopathy	1.25	0.57	4.75	1	0.029	3.48	1.13–10.71
Need for an intensive care unit	2.03	0.58	12.21	1	0.001	7.64	2.44–23.92
Need for ventilatory support							
Invasive	0.91	0.65	1.97	1	0.160	2.49	0.70–8.92
Non-invasive	−0.78	0.59	1.75	1	0186	0.6	0.14–1.46
Not performed (reference)			8.20	2	0.017		
Constant	−4.15	0.73	32,54	1	0.001	0.01	

95%CI, 95% confidence interval; df, degrees of freedom; SE, standard error; OR, odds ratio. The following markers were included in the model: viral infection (bocavirus, metapneumovirus, rhinovirus, and respiratory syncytial virus), age, race, comorbidities (cardiopathy, hepatic disorder, diabetes mellitus, and immunodepression), need for an intensive care unit, and need for mechanical ventilatory support. The multivariable analysis was done using the Binary Logistic Regression model with the Backward Stepwise method. Markers with *p* ≤ 0.05 in the bivariate analysis were included in the regression model. The dependent variable was the health outcome (death or recovery—hospital discharge). The alpha error of 0.05 was considered in the multivariable analyses carried out in the study.

a Other races included the patients self-declared as Black people, Asian individuals, Mixed individuals (*Pardos*), and Indigenous peoples. The data were collected in the Open-Data-SUS (https://opendatasus.saude.gov.br/). The data comprised the period from December 19, 2019, to April 06, 2023—three years since the beginning of the coronavirus disease (COVID)-19 pandemic in Brazil.

## 4 Discussion

In our study, we observed that the co-detection of other respiratory viruses in hospitalized patients due to influenza occurred, mainly, by the RSV and rhinovirus in patients infected with the influenza A virus. In our analysis, two viruses (bocavirus and rhinovirus) were associated with an increased chance of death. Several other factors were also associated with an increased chance of death, such as race, the presence of comorbidity, the need for ICU treatment, and the need for ventilatory support. The main symptoms involved respiratory complications and the mortality rate was 6.9%.

The H3N2 lineage of the influenza A virus presented the highest prevalence in our study, being that this particular lineage was described in co-detection with other influenza A and B lineages (Gregianini et al., 2019). The presence of different viral lineages in the same host can be responsible for genetic rearrangements and result in new variants with greater adaptive resistance, which can culminate in challenges to infection control (Nelson and Vincent, 2015). Furthermore, the co-detection rate is variable and depends on the enrolled population, the age of the individuals, and social, clinical, and demographic factors. In this way, the literature describes such a wide range of co-detection, varying from 7.3% in the United States of America and 61.8% in Spain (Echenique et al., 2013; Martínez-Roig et al., 2015).

In our study, most of the respiratory viruses were most common in children, such as rhinovirus, RSV, and adenovirus, which is per the literature (Zhang et al., 2021; Dallmeyer et al., 2024; Dias et al., 2024). Classically, respiratory viruses have always been a greater threat to young children, due to the immune system that is still developing and smaller airways and lungs, which can facilitate virus infection (CDC, 2024b), in fact, most of these viruses are transmitted by poor hygiene or even contact with someone who was already sick; that is why older people can protect themselves by washing hands or other hygiene measures, thus decreasing the impact of transmission.

The profile of the respiratory viruses that were co-detected with influenza was similar to those described in the literature. Depending on the co-detection profile, the patient’s outcome might be more severe (Chauhan and Slamon, 2017; Noyola et al., 2019). In Brazil, in turn, few studies have evaluated respiratory viral co-detection (Martins Júnior et al., 2014; Canela et al., 2018; Costa et al., 2022; Moreira et al., 2023). Canela et al. (2018) evaluated 71 samples of patients up to 18 years of age who were treated in the ICU, the influenza virus being the most common (43%), followed by the rhinovirus (41%). The co-detection occurred in almost 22% of the samples, being described as the co-detection of H1N1 and rhinovirus/enterovirus, RSV, metapneumovirus, and seasonal influenza virus, however, it was not possible to describe the impact of this viral profile on the outcome (Canela et al., 2018). On the other hand, Costa et al. (2022) described a more severe clinical profile of hospitalized patients with viral co-detection (Costa et al., 2022). Also, Martins Júnior et al. (2014) observed that in outpatients, the viral co-detection rate is low (3.7%), and if presented, most of the co-detection was associated with RSV (Martins Júnior et al., 2014). Nevertheless, the literature is still controversial regarding the impact of viral co-detection on clinical outcomes and what the effector role is in this outcome according to the different etiological agents detected and the ecological relationship between them.

Regarding seasonality, in our study, most of the viruses were detected in December and January, months with warmer temperatures in the South Hemisphere. Although studies described the higher incidence of the respiratory virus in colder seasons (Viegas et al., 2004; Weigl et al., 2007; Ambrosioni et al., 2014, pp. 2011–2012; García-Arroyo et al., 2022), in Brazil, previous studies observed a different trend in seasonality in respiratory virus (Gardinassi et al., 2012; Vianna et al., 2021), in which a peak of incidence was observed in spring and autumn. In our study, since most of the data were acquired from the Brazilian Ministry of Health dataset, tests were completely at the discretion of the attending physicians and availability in the Health Institutions, which could reflect a pseudo-increase in warmer temperature seasons, such as January and December. One might also speculate that the lower humidity in Brazil, especially in dry regions such as the *cerrado*, contributed to a higher incidence of respiratory viruses, since their incidence appears to increase in low-humidity regions climate (Gardinassi et al., 2012; Hofmann et al., 2023).

In general, in our study, the co-detection of other respiratory viruses than influenza was low; however, the literature seems to be controversial. A Brazilian study performed by Dias et al. (2024) observed co-detection among respiratory viruses that ranged from 0.3–1.3% (Dias et al., 2024), while in the study by De Paulis et al. (2011), 31% of the patients presented with co-detection (De Paulis et al., 2011). In the same way, co-detection also varies according to region; for example, in Cameroon, co-detection was around 6.9% (Moumbeket Yifomnjou et al., 2023, pp. 2020–2021), which is similar to countries such as France (6.5%) (Le Hingrat et al., 2021). Similarly, the effect of respiratory virus co-detections on patients’ outcomes remains unclear. For example, some studies did not describe an association between co-detection of other respiratory viruses and worse outcomes (Cilla et al., 2008; De Paulis et al., 2011), in contrast, Semple et al. (2005) observed an increased risk of ICU for mechanical ventilation in pediatric patients with metapneumovirus and RSV (Semple et al., 2005). Since the real effect of co-detection on the worst outcomes is still not clear in the literature, further studies are necessary to evaluate the impact of viral co-detections in hospitalized patients.

The respiratory viruses have a seasonal profile, therefore mutual infections may be the result of this natural flow (Rath et al., 2017). The interactions between respiratory viruses may present a different response depending on the first viral infection (Godinho et al., 2016; Piret and Boivin, 2022). In this context, the first virus results in a positive response (synergism or additive effect) or a negative response (antagonism effect among the virus) (Piret and Boivin, 2022). Similarly, to the outcome, severity is represented as a broad phenotypic spectrum, which, consequently, denotes the search for medical support through the clinical condition. This directive search may be a conditional factor for the diagnosis of the viral profile most commonly associated with worse clinical outcomes and which may generate underreporting of milder clinical phenotypic (Szymański et al., 2017).

The juvenile was the most affected in our study; however, it was not associated with an enhanced chance of death. Younger individuals have an immature immune system, which makes these individuals more vulnerable to viral infections (Debiaggi et al., 2012; Cebey-López et al., 2015). The lack of acquired immunity to the different viruses might also enhance the co-detection (Cieślak et al., 2018). The biological and environmental markers, such as experience in educational and daycare centers, can culminate in an increase in viral infection in this age group and thus in co-detection (Cieślak et al., 2018; Kurskaya et al., 2018). Other races, such as Black people, Indigenous peoples, and mixed individuals (*Pardos*), on the other hand, were associated with an enhanced chance of death, which is similar to previous studies (Sansone et al., 2022a; CDC, 2024a). One might speculate that these people have less access to healthcare, which might culminate in delayed hospitalization and worse outcomes (Williams and Rucker, 2000; Racial and income inequalities in access to healthcare in Brazilian cities—ScienceDirect, 2024).

The death rate in the present study was relatively low considering that all patients were hospitalized for influenza virus infection. However, the literature lacks good evidence on the real impact of multiple viral infections on patient outcomes, possibly due to the low rates of diagnosis of these infections. However, the main findings favor a greater assertiveness of severity in the face of viral infections in line with those of bacterial origin (Rice et al., 2012; Diaz et al., 2016). Although the literature is uncertain, in our findings, some viruses and comorbidities were described as risk factors for death and, given the above, other studies should be listed to validate our findings (Rath et al., 2017; Kurskaya et al., 2018; Barahimi et al., 2023).

The control over viral infections in countries such as Brazil is of paramount importance for local epidemiology. Although the literature contradicts the real effect of co-detection on more serious outcomes in hospitalized patients, testing for pathogens that have specific therapy, such as the influenza virus, appears to be minimally reasonable (Wishaupt et al., 2017; Costa et al., 2022; Ambrożej et al., 2024). From an epidemiological point of view, mass testing of patients is difficult, therefore, it is extremely important to take into account not only clinical and demographic characteristics, such as age and clinical presentation, but also to take into account periods of the year, as most viruses have their seasonality (Hall, 2001; McAdam et al., 2004; Hermos et al., 2010; Ginocchio and McAdam, 2011).

Although our study mainly dealt with patients with a positive result for influenza and the co-detection of other respiratory viruses, undoubtedly, the COVID-19 pandemic played an important role in the dynamics of surveillance of flu-like syndromes. A recent report observed 21 surveillance influenza virus infections and COVID-19 surveillance systems from different countries (including Brazil) from 2021–2022 (Staadegaard et al., 2023). In most of these countries, the authors observed decreased influenza activity and a decreased number of samples tested at least temporally (Adlhoch et al., 2021; Staadegaard et al., 2023; End-to-end integration of SARS-CoV-2 and influenza sentinel surveillance: compendium of country approaches, 2024). Several reasons might explain this particular finding, such as structural changes in the surveillance system and decreased syndromic consultations (Adlhoch et al., 2021; Staadegaard et al., 2023). One might speculate that the pandemic limited the circulation of the influenza virus as shown in previous research (Adlhoch et al., 2021; Staadegaard et al., 2023), in which the rates of rhinovirus, influenza, RSV, and adenovirus decreased in 2020 compared to 2019 perhaps due to quarantine and social isolation (Kuitunen et al., 2020; Trenholme et al., 2021), highlighting the importance of COVID-19 not only as a disease itself but also disrupting other virus seasonality.

Public data surveillance of respiratory viruses is also extremely relevant worldwide, as its use has the potential to detect possible epidemics and upcoming outbreaks of already known viruses or even new viruses, as was the case with SARS-CoV-2 (Hashimoto et al., 2000; Morse, 2012; Maddah et al., 2023). Since 2009, in response to the H1N1 pandemic, Brazil has developed SIVEP-Gripe to control cases and possible viral outbreaks (da Silva et al., 2022). Notifications are made by clinics, hospitals, and also emergency departments, both public and private, which means that all strata of the population are covered (Sansone et al., 2022b; Martins et al., 2023; Palamim et al., 2023). Unfortunately, the specific virus evaluation of these patients may be underdiagnosed, as was the case with COVID-19, which can make it difficult to implement public health policies for the surveillance and control of infections.

### 4.1 Limitations

The study has limitations that require caution when interpreting the results. The data source, coming from the Open-Data-SUS platform, may have representativeness and accuracy restrictions, which affect the generalization of the findings. Collecting data exclusively from hospitalized patients introduces potential selection bias, limiting the representativeness of the diversity of influenza virus infection in the general population. Generalization to other populations is restricted by the specificity of the Brazilian context during the COVID-19 pandemic. The definition of comorbidities may lack uniformity and the lack of exploration of socioeconomic variables and the history of vaccination may affect understanding of the determinants of death risk. There is no information on the diagnoses of important respiratory virus infections responsible for common colds, such as those caused by OC43 and NL63 strains, since the institutions that provided the Open-Data-SUS did not test for these specific strains in Brazil. Additionally, only a small portion of hospitalized patients in Brazil have been tested for several respiratory viruses. Laboratory tests occurred randomly depending on medical requests, availability of tests in health institutions, and access to tests by patients. Therefore, one of the main limitations of the study is the difficulty in providing accurate information on the temporal analysis of infections. Furthermore, there was an outbreak of influenza virus infection at the end of 2021 which may have helped to increase cases in the months surrounding the outbreak, mainly the summer season. We excluded patients with a positive SARS-CoV-2 diagnosis since we were only interested in patients with influenza in the pandemic era. In addition, several studies were performed to understand co-detection/co-infection of respiratory viruses including SARS-CoV-2 (Kim et al., 2020; Swets et al., 2022; Morales-Jadán et al., 2023); however, we tried to understand the burden of the co-detection between influenza virus and other respiratory viruses during the COVID-19 pandemic.

## 5 Conclusion

The co-detection between the influenza virus and other respiratory viruses occurred, mainly with RSV, rhinovirus, and adenovirus being more common in men, white people, and in the juvenile phase. Co-detection of influenza virus with bocavirus and metapneumovirus was associated with an increased chance of death. Other factors such as race, heart disease, and the need for ICU were also associated with a higher chance of death.

## Data Availability

The raw data supporting the conclusions of this article will be made available by the authors, without undue reservation.

## Footnotes

^1^ https://opendatasus.saude.gov.br/

## References

[ref1] Adlhoch C. Sneiderman M. Martinuka O. Melidou A. Bundle N. Fielding J.. (2021). Spotlight influenza: the 2019/20 influenza season and the impact of COVID-19 on influenza surveillance in the WHO European region. Euro Surveill. 26:2100077. doi: 10.2807/1560-7917.ES.2021.26.40.2100077, PMID: 34622760 PMC8511754

[ref2] Alexander P. Dobrovolny H. M. (2022). Treatment of respiratory viral coinfections. Epidemiologia 3, 81–96. doi: 10.3390/epidemiologia3010008, PMID: 36417269 PMC9620919

[ref3] Ambrosioni J. Bridevaux P.-O. Wagner G. Mamin A. Kaiser L. (2014). Epidemiology of viral respiratory infections in a tertiary care Centre in the era of molecular diagnosis, Geneva, Switzerland, 2011–2012. Clin. Microbiol. Infect. 20, O578–O584. doi: 10.1111/1469-0691.12525, PMID: 24382326 PMC7128668

[ref4] Ambrożej D. Orzołek I. Makrinioti H. Castro-Rodriguez J. A. Camargo C. A. Hasegawa K.. (2024). Association of respiratory virus types with clinical features in bronchiolitis: implications for virus testing strategies. A systematic review and meta-analysis. Paediatr. Respir. Rev. 49, 34–42. doi: 10.1016/j.prrv.2023.09.003, PMID: 37743159

[ref5] Ao T. McCracken J. P. Lopez M. R. Bernart C. Chacon R. Moscoso F.. (2019). Hospitalization and death among patients with influenza, Guatemala, 2008–2012. BMC Public Health 19:463. doi: 10.1186/s12889-019-6781-6, PMID: 32326933 PMC6696630

[ref6] Baqui P. Bica I. Marra V. Ercole A. van der Schaar M. (2020). Ethnic and regional variations in hospital mortality from COVID-19 in Brazil: a cross-sectional observational study. Lancet Glob. Health 8, e1018–e1026. doi: 10.1016/S2214-109X(20)30285-0, PMID: 32622400 PMC7332269

[ref7] Barahimi E. Azad M. H. Hesarooeyeh Z. G. Hafshejani N. H. Defaee S. Seddighi N. (2023). Late diagnosis of respiratory syncytial virus and influenza co-infection during coronavirus disease 2019 pandemic: a case report. J. Med. Case Rep. 17:437. doi: 10.1186/s13256-023-04187-3, PMID: 37864237 PMC10589917

[ref8] Boschiero M. N. Duarte A. Palamim C. V. C. Alvarez A. E. Mauch R. M. Marson F. A. L. (2022a). Frequency of respiratory pathogens other than SARS-CoV-2 detected during COVID-19 testing. Diagn. Microbiol. Infect. Dis. 102:115576. doi: 10.1016/j.diagmicrobio.2021.115576, PMID: 34800846 PMC8531239

[ref9] Boschiero M. N. Palamim C. V. C. Ortega M. M. Marson F. A. L. (2022b). Clinical characteristics and comorbidities of COVID-19 in unvaccinated patients with down syndrome: first year report in Brazil. Hum. Genet. 141, 1887–1904. doi: 10.1007/s00439-022-02468-3, PMID: 35763088 PMC9244024

[ref10] Canela L. N. P. Magalhães-Barbosa M. C. Raymundo C. E. Carney S. Siqueira M. M. Prata-Barbosa A.. (2018). Viral detection profile in children with severe acute respiratory infection. Braz. J. Infect. Dis. 22, 402–411. doi: 10.1016/j.bjid.2018.09.001, PMID: 30365924 PMC7138071

[ref11] CDC (2024a). Racial and ethnic minority groups. Centers for Disease Control and Prevention. Available at: https://www.cdc.gov/flu/highrisk/disparities-racial-ethnic-minority-groups.html (Accessed September 6, 2024).

[ref12] CDC (2024b). Respiratory viruses and young children. Respiratory illnesses. Available at: https://www.cdc.gov/respiratory-viruses/risk-factors/young-children.html (Accessed September 6, 2024).

[ref13] Cebey-López M. Herberg J. Pardo-Seco J. Gómez-Carballa A. Martinón-Torres N. Salas A.. (2015). Viral co-infections in pediatric patients hospitalized with lower tract acute respiratory infections. PLoS One 10:e0136526. doi: 10.1371/journal.pone.0136526, PMID: 26332375 PMC4558027

[ref14] Chauhan J. C. Slamon N. B. (2017). The impact of multiple viral respiratory infections on outcomes for critically ill children. Pediatr. Crit. Care Med. 18, e333–e338. doi: 10.1097/PCC.0000000000001232, PMID: 28628546

[ref15] Choe Y. J. Park S. Michelow I. C. (2020). Co-seasonality and co-detection of respiratory viruses and bacteraemia in children: a retrospective analysis. Clin. Microbiol. Infect. 26, 1690.e5–1690.e8. doi: 10.1016/j.cmi.2020.09.006, PMID: 32919073 PMC7481115

[ref16] Chu S.-E. Seak C.-J. Su T.-H. Chaou C.-H. Tseng H.-J. Li C.-H. (2020). Prognostic accuracy of SIRS criteria and qSOFA score for in-hospital mortality among influenza patients in the emergency department. BMC Infect. Dis. 20:385. doi: 10.1186/s12879-020-05102-7, PMID: 32471385 PMC7256917

[ref17] Cieślak K. Kowalczyk D. Szymański K. Hallmann-Szelińska E. Brydak L. B. (2018). Influenza and influenza-like viruses: frequent infections in children under 14 years of age during the 2016/2017 epidemic season. Adv. Exp. Med. Biol. 1114, 83–87. doi: 10.1007/5584_2018_229, PMID: 30117125 PMC7124040

[ref18] Cilla G. Oñate E. Perez-Yarza E. G. Montes M. Vicente D. Perez-Trallero E. (2008). Viruses in community-acquired pneumonia in children aged less than 3 years old: high rate of viral coinfection. J. Med. Virol. 80, 1843–1849. doi: 10.1002/jmv.21271, PMID: 18712820 PMC7166914

[ref19] Corouraça|Educa|Jovens—IBGE (2024). Available at: https://educa.ibge.gov.br/jovens/conheca-o-brasil/populacao/18319-cor-ou-raca.html (Accessed January 5, 2024).

[ref20] Costa L. F. Da Silveira H. L. Queiróz D. A. O. Mantese O. C. Yokosawa J. (2022). Respiratory virus infections in hospitalized and non-hospitalized children: determinants of severe course of the disease. J. Infect. Dev. Ctries. 16, 196–205. doi: 10.3855/jidc.15117, PMID: 35192538

[ref21] da Silva A. D. da Veiga A. B. G. Cruz O. G. Bastos L. S. Gomes M. F. (2022). Severe acute respiratory infection surveillance in Brazil: the role of public, private and philanthropic healthcare units. Health Policy Plan. 37, 1075–1085. doi: 10.1093/heapol/czac050, PMID: 35766892 PMC9384390

[ref22] Dallmeyer L. K. Schüz M. L. Fragkou P. C. Omony J. Krumbein H. Dimopoulou D.. (2024). Epidemiology of respiratory viruses among children during the SARS-CoV-2 pandemic: a systematic review and meta-analysis. Int. J. Infect. Dis. 138, 10–18. doi: 10.1016/j.ijid.2023.10.023, PMID: 37951460

[ref23] De Paulis M. Gilio A. E. Ferraro A. A. Ferronato A. E. Sacramento P. R. Botosso V. F.. (2011). Severity of viral coinfection in hospitalized infants with respiratory syncytial virus infection. J. Pediatr. 87, 307–313. doi: 10.2223/JPED.2100 21655684

[ref24] de Souza W. M. Buss L. F. Candido D. Carrera J.-P. Li S. Zarebski A. E.. (2020). Epidemiological and clinical characteristics of the COVID-19 epidemic in Brazil. Nat. Hum. Behav. 4, 856–865. doi: 10.1038/s41562-020-0928-4 32737472

[ref25] Debiaggi M. Canducci F. Ceresola E. R. Clementi M. (2012). The role of infections and coinfections with newly identified and emerging respiratory viruses in children. Virol. J. 9:247. doi: 10.1186/1743-422X-9-247, PMID: 23102237 PMC3573994

[ref26] Dias C. S. Diniz L. M. Oliveira M. C. L. Simoes E Silva A. C. Colosimo E. A. Mak R. H.. (2024). Outcomes of SARS-CoV-2 and seasonal viruses among children hospitalized in Brazil. Pediatrics 153:e2023064326. doi: 10.1542/peds.2023-064326, PMID: 38213278

[ref27] Diaz M. H. Cross K. E. Benitez A. J. Hicks L. A. Kutty P. Bramley A. M.. (2016). Identification of bacterial and viral Codetections with Mycoplasma pneumoniae using the TaqMan Array card in patients hospitalized with community-acquired pneumonia. Open forum. Infect. Dis. 3:ofw071. doi: 10.1093/ofid/ofw071, PMID: 27191004 PMC4867659

[ref28] Dyussenbayev A. (2017). Age periods of human life. Adv. Soc. Sci. Res. J. 4:2924. doi: 10.14738/assrj.46.2924

[ref29] Echenique I. A. Chan P. A. Chapin K. C. Andrea S. B. Fava J. L. Mermel L. A. (2013). Clinical characteristics and outcomes in hospitalized patients with respiratory viral co-infection during the 2009 H1N1 influenza pandemic. PLoS One 8:e60845. doi: 10.1371/journal.pone.0060845, PMID: 23585856 PMC3622008

[ref30] End-to-end integration of SARS-CoV-2 and influenza sentinel surveillance: compendium of country approaches (2024). Available at: https://www.who.int/publications/i/item/9789240056701 (Accessed September 4, 2024).

[ref31] García-Arroyo L. Prim N. Del Cuerpo M. Marín P. Roig M. C. Esteban M.. (2022). Prevalence and seasonality of viral respiratory infections in a temperate climate region: a 24-year study (1997–2020). Influenza Other Respir. Viruses 16, 756–766. doi: 10.1111/irv.12972, PMID: 35170253 PMC9178050

[ref32] Gardinassi L. G. Simas P. V. M. Salomão J. B. Durigon E. L. Trevisan D. M. Z. Cordeiro J. A.. (2012). Seasonality of viral respiratory infections in southeast of Brazil: the influence of temperature and air humidity. Braz. J. Microbiol. 43, 98–108. doi: 10.1590/S1517-838220120001000011, PMID: 24031808 PMC3768995

[ref33] Ginocchio C. C. McAdam A. J. (2011). Current Best practices for respiratory virus testing. J. Clin. Microbiol. 49, S44–S48. doi: 10.1128/JCM.00698-11

[ref34] Godinho C. A. Yardley L. Marcu A. Mowbray F. Beard E. Michie S. (2016). Increasing the intent to receive a pandemic influenza vaccination: testing the impact of theory-based messages. Prev. Med. 89, 104–111. doi: 10.1016/j.ypmed.2016.05.025, PMID: 27235605

[ref35] Gregianini T. S. Varella I. R. S. Fisch P. Martins L. G. Veiga A. B. G. (2019). Dual and triple infections with influenza A and B viruses: a case-control study in southern Brazil. J. Infect. Dis. 220, 961–968. doi: 10.1093/infdis/jiz221, PMID: 31125400

[ref36] Hall C. B. (2001). Respiratory syncytial virus and parainfluenza virus. N. Engl. J. Med. 344, 1917–1928. doi: 10.1056/NEJM200106213442507 11419430

[ref37] Hashimoto S. Murakami Y. Taniguchi K. Nagai M. (2000). Detection of epidemics in their early stage through infectious disease surveillance. Int. J. Epidemiol. 29, 905–910. doi: 10.1093/ije/29.5.905 11034976

[ref38] Hermos C. R. Vargas S. O. McAdam A. J. (2010). Human Metapneumovirus. Clin. Lab. Med. 30, 131–148. doi: 10.1016/j.cll.2009.10.002, PMID: 20513544 PMC7115734

[ref39] Hofmann G. S. Silva R. C. Weber E. J. Barbosa A. A. Oliveira L. F. B. Alves R. J. V.. (2023). Changes in atmospheric circulation and evapotranspiration are reducing rainfall in the Brazilian Cerrado. Sci. Rep. 13:11236. doi: 10.1038/s41598-023-38174-x, PMID: 37433851 PMC10336145

[ref40] Iuliano A. D. Roguski K. M. Chang H. H. Muscatello D. J. Palekar R. Tempia S.. (2018). Estimates of global seasonal influenza-associated respiratory mortality: a modelling study. Lancet 391, 1285–1300. doi: 10.1016/S0140-6736(17)33293-2, PMID: 29248255 PMC5935243

[ref41] Kim D. Quinn J. Pinsky B. Shah N. H. Brown I. (2020). Rates of co-infection between SARS-CoV-2 and other respiratory pathogens. JAMA 323, 2085–2086. doi: 10.1001/jama.2020.6266, PMID: 32293646 PMC7160748

[ref42] Kuitunen I. Artama M. Mäkelä L. Backman K. Heiskanen-Kosma T. Renko M. (2020). Effect of social distancing due to the COVID-19 pandemic on the incidence of viral respiratory tract infections in children in Finland during early 2020. Pediatr. Infect. Dis. J. 39, e423–e427. doi: 10.1097/INF.0000000000002845, PMID: 32773660

[ref43] Kurskaya O. Ryabichenko T. Leonova N. Shi W. Bi H. Sharshov K.. (2018). Viral etiology of acute respiratory infections in hospitalized children in Novosibirsk City, Russia (2013–2017). PLoS One 13:e0200117. doi: 10.1371/journal.pone.0200117, PMID: 30226876 PMC6143185

[ref44] Lafond K. E. Porter R. M. Whaley M. J. Suizan Z. Ran Z. Aleem M. A.. (2021). Global burden of influenza-associated lower respiratory tract infections and hospitalizations among adults: a systematic review and meta-analysis. PLoS Med. 18:e1003550. doi: 10.1371/journal.pmed.1003550, PMID: 33647033 PMC7959367

[ref45] Le Hingrat Q. Bouzid D. Choquet C. Laurent O. Lescure F.-X. Timsit J.-F.. (2021). Viral epidemiology and SARS-CoV-2 co-infections with other respiratory viruses during the first COVID-19 wave in Paris, France. Influenza Other Respir. Viruses 15, 425–428. doi: 10.1111/irv.12853, PMID: 33817971 PMC8189235

[ref46] Maddah N. Verma A. Almashmoum M. Ainsworth J. (2023). Effectiveness of public health digital surveillance Systems for Infectious Disease Prevention and Control at mass gatherings: systematic review. J. Med. Internet Res. 25:e44649. doi: 10.2196/44649, PMID: 37204833 PMC10238952

[ref47] Martínez-Roig A. Salvadó M. Caballero-Rabasco M. A. Sánchez-Buenavida A. López-Segura N. Bonet-Alcaina M. (2015). Viral coinfection in childhood respiratory tract infections. Arch. Bronconeumol. 51, 5–9. doi: 10.1016/j.arbres.2014.01.018 24666712 PMC7127166

[ref48] Martin-Loeches I. Lemiale V. Geoghegan P. McMahon M. A. Pickkers P. Soares M.. (2019). Influenza and associated co-infections in critically ill immunosuppressed patients. Crit. Care 23:152. doi: 10.1186/s13054-019-2425-6, PMID: 31046842 PMC6498695

[ref49] Martins Júnior R. B. Carney S. Goldemberg D. Bonine L. Spano L. C. Siqueira M.. (2014). Detection of respiratory viruses by real-time polymerase chain reaction in outpatients with acute respiratory infection. Mem. Inst. Oswaldo Cruz 109, 716–721. doi: 10.1590/0074-0276140046, PMID: 25317699 PMC4238762

[ref50] Martins J. P. Siqueira B. A. Sansone N. M. S. Marson F. A. L. (2023). COVID-19 in Brazil: a three-year update. Diagn. Microbiol. Infect. Dis. 2023:116074. doi: 10.1016/j.diagmicrobio.2023.116074 37729718

[ref51] McAdam A. J. Hasenbein M. E. Feldman H. A. Cole S. E. Offermann J. T. Riley A. M.. (2004). Human metapneumovirus in children tested at a tertiary-care hospital. J. Infect. Dis. 190, 20–26. doi: 10.1086/421120, PMID: 15195239

[ref52] Ministério da Saúde (2024). Ministério da Saúde. Available at: https://www.gov.br/saude/pt-br/pagina-inicial (Accessed September 3, 2024).

[ref53] Morales-Jadán D. Muslin C. Viteri-Dávila C. Coronel B. Castro-Rodríguez B. Vallejo-Janeta A. P.. (2023). Coinfection of SARS-CoV-2 with other respiratory pathogens in outpatients from Ecuador. Front. Public Health 11:1264632. doi: 10.3389/fpubh.2023.1264632, PMID: 37965509 PMC10641819

[ref54] Moreira A. L. E. da Silva P. A. N. Assunção L. Santos M. O. Ito C. R. M. de Araújo K. M.. (2023). Profile analysis of emerging respiratory virus in children. Eur. J. Clin. Microbiol. Infect. Dis. 42, 873–882. doi: 10.1007/s10096-023-04615-8, PMID: 37160574 PMC10169160

[ref55] Morse S. S. (2012). Public health surveillance and infectious disease detection. Biosecur. Bioterror. 10, 6–16. doi: 10.1089/bsp.2011.0088 22455675

[ref56] Moumbeket Yifomnjou M. H. Monamele G. C. Njankouo-Ripa M. Fatawou Modiyinji A. Ngoupo P. A. Boyomo O.. (2023). Viral co-infection with human respiratory syncytial virus in suspected acute and severe respiratory tract infections during COVID-19 pandemic in Yaoundé, Cameroon, 2020–2021. Influenza Other Respir. Viruses 17:e13131. doi: 10.1111/irv.13131, PMID: 36991539 PMC10060445

[ref57] Nelson M. I. Vincent A. L. (2015). Reverse zoonosis of influenza to swine: new perspectives on the human-animal interface. Trends Microbiol. 23, 142–153. doi: 10.1016/j.tim.2014.12.002, PMID: 25564096 PMC4348213

[ref58] Noyola D. E. Hunsberger S. Valdés Salgado R. Powers J. H. Galindo-Fraga A. Ortiz-Hernández A. A.. (2019). Comparison of rates of hospitalization between single and dual virus detection in a Mexican cohort of children and adults with influenza-like illness. Open Forum Infect. Dis. 6:ofz424. doi: 10.1093/ofid/ofz424, PMID: 31696140 PMC6824528

[ref59] Palamim C. V. C. Siqueira B. A. Boschiero M. N. Marson F. A. L. (2023). Increase in COVID-19 underreporting among 3,282,337 Brazilian hospitalized patients due to SARS: a 3-year report and a major concern for health authorities. Travel Med. Infect. Dis. 54:102616. doi: 10.1016/j.tmaid.2023.102616, PMID: 37442515

[ref60] Pinky L. Dobrovolny H. M. (2016). Coinfections of the respiratory tract: viral competition for resources. PLoS One 11:e0155589. doi: 10.1371/journal.pone.0155589, PMID: 27196110 PMC4873262

[ref61] Piret J. Boivin G. (2022). Viral interference between respiratory viruses. Emerg. Infect. Dis. 28, 273–281. doi: 10.3201/eid2802.211727, PMID: 35075991 PMC8798701

[ref62] Racial and income inequalities in access to healthcare in Brazilian cities—ScienceDirect (2024). Available at: https://www.sciencedirect.com/science/article/pii/S2214140523001585 (Accessed September 6, 2024).

[ref63] Rath B. Conrad T. Myles P. Alchikh M. Ma X. Hoppe C.. (2017). Influenza and other respiratory viruses: standardizing disease severity in surveillance and clinical trials. Expert Rev. Anti-Infect. Ther. 15, 545–568. doi: 10.1080/14787210.2017.1295847, PMID: 28277820 PMC7103706

[ref64] Rice T. W. Rubinson L. Uyeki T. M. Vaughn F. L. John B. B. Miller R. R.. (2012). Critical illness from 2009 pandemic influenza a virus and bacterial coinfection in the United States. Crit. Care Med. 40, 1487–1498. doi: 10.1097/CCM.0b013e3182416f23, PMID: 22511131 PMC3653183

[ref65] Sansone N. M. S. Boschiero M. N. Marson F. A. L. (2022b). Epidemiologic profile of severe acute respiratory infection in Brazil during the COVID-19 pandemic: an epidemiological study. Front. Microbiol. 13:911036. doi: 10.3389/fmicb.2022.911036, PMID: 35854935 PMC9288583

[ref66] Sansone N. M. Boschiero M. N. Valencise F. E. Palamim C. V. Marson F. A. (2022a). Characterization of demographic data, clinical signs, comorbidities, and outcomes according to the race in hospitalized individuals with COVID-19 in Brazil: an observational study. J. Glob. Health 12:5027. doi: 10.7189/jogh.12.05027, PMID: 35871427 PMC9309002

[ref67] Semple M. G. Cowell A. Dove W. Greensill J. McNamara P. S. Halfhide C.. (2005). Dual infection of infants by human metapneumovirus and human respiratory syncytial virus is strongly associated with severe bronchiolitis. J. Infect. Dis. 191, 382–386. doi: 10.1086/426457, PMID: 15633097 PMC7109698

[ref68] Staadegaard L. Del Riccio M. Wiegersma S. El Guerche-Séblain C. Dueger E. Akçay M.. (2023). The impact of the SARS-CoV-2 pandemic on global influenza surveillance: insights from 18 National Influenza Centers based on a survey conducted between November 2021 and march 2022. Influenza Other Respir. Viruses 17:e13140. doi: 10.1111/irv.13140, PMID: 37180840 PMC10173050

[ref69] Sullivan K. M. Dean A. Soe M. M. (2009). OpenEpi: a web-based epidemiologic and statistical calculator for public health. Public Health Rep. 124, 471–474, PMID: 19445426 10.1177/003335490912400320 PMC2663701

[ref70] Swets M. C. Russell C. D. Harrison E. M. Docherty A. B. Lone N. Girvan M.. (2022). SARS-CoV-2 co-infection with influenza viruses, respiratory syncytial virus, or adenoviruses. Lancet 399, 1463–1464. doi: 10.1016/S0140-6736(22)00383-X, PMID: 35344735 PMC8956294

[ref71] Sze S. Pan D. Nevill C. R. Gray L. J. Martin C. A. Nazareth J.. (2020). Ethnicity and clinical outcomes in COVID-19: a systematic review and meta-analysis. EClinicalMedicine 29:100630. doi: 10.1016/j.eclinm.2020.100630, PMID: 33200120 PMC7658622

[ref72] Szymański K. Cieślak K. Kowalczyk D. Brydak L. B. (2017). Co-infection with influenza viruses and influenza-like virus during the 2015/2016 epidemic season. Adv. Exp. Med. Biol. 968, 7–12. doi: 10.1007/5584_2016_182, PMID: 28181195 PMC7122344

[ref73] The burden of Influenza (2024). Available at: https://www.who.int/news-room/feature-stories/detail/the-burden-of-influenza (Accessed May 16, 2024).

[ref74] Trenholme A. A. Best E. J. Vogel A. M. Stewart J. M. Miller C. J. Lennon D. R. (2017). Respiratory virus detection during hospitalisation for lower respiratory tract infection in children under 2 years in South Auckland, New Zealand. J. Paediatr. Child Health 53, 551–555. doi: 10.1111/jpc.13529, PMID: 28430397

[ref75] Trenholme A. Webb R. Lawrence S. Arrol S. Taylor S. Ameratunga S.. (2021). COVID-19 and infant hospitalizations for seasonal respiratory virus infections, New Zealand, 2020. Emerg. Infect. Dis. 27, 641–643. doi: 10.3201/eid2702.204041, PMID: 33263515 PMC7853573

[ref76] Vianna L. A. Siqueira M. M. Volpini L. P. B. Louro I. D. Resende P. C. (2021). Seasonality, molecular epidemiology, and virulence of respiratory syncytial virus (RSV): a perspective into the Brazilian influenza surveillance program. PLoS One 16:e0251361. doi: 10.1371/journal.pone.0251361, PMID: 34003843 PMC8130917

[ref77] Viegas M. Barrero P. R. Maffey A. F. Mistchenko A. S. (2004). Respiratory viruses seasonality in children under five years of age in Buenos Aires, Argentina: a five-year analysis. J. Infect. 49, 222–228. doi: 10.1016/j.jinf.2003.10.006, PMID: 15337339

[ref78] Weigl J. A. I. Puppe W. Meyer C. U. Berner R. Forster J. Schmitt H. J.. (2007). Ten years’ experience with year-round active surveillance of up to 19 respiratory pathogens in children. Eur. J. Pediatr. 166, 957–966. doi: 10.1007/s00431-007-0496-x, PMID: 17569085 PMC7087302

[ref79] Williams D. R. Rucker T. D. (2000). Understanding and addressing racial disparities in health care. Health Care Financ. Rev. 21, 75–90, PMID: 11481746 PMC4194634

[ref80] Wishaupt J. O. van der Ploeg T. de Groot R. Versteegh F. G. A. Hartwig N. G. (2017). Single-and multiple viral respiratory infections in children: disease and management cannot be related to a specific pathogen. BMC Infect. Dis. 17:62. doi: 10.1186/s12879-016-2118-6, PMID: 28077074 PMC5225597

[ref81] Yan X. Li K. Lei Z. Luo J. Wang Q. Wei S. (2023). Prevalence and associated outcomes of coinfection between SARS-CoV-2 and influenza: a systematic review and meta-analysis. Int. J. Infect. Dis. 136, 29–36. doi: 10.1016/j.ijid.2023.08.021, PMID: 37648094

[ref82] Zhang Y. Qiao L. Yao J. Yu N. Mu X. Huang S.. (2021). Epidemiological and clinical characteristics of respiratory viruses in 4403 pediatric patients from multiple hospitals in Guangdong, China. BMC Pediatr 21, 1–10. doi: 10.1186/s12887-021-02759-0 34140022 PMC8212487

