# Supplementary material for: Viral co-detection of influenza virus and other respiratory viruses in hospitalized Brazilian patients during the first three years of the coronavirus disease (COVID)-19 pandemic: an epidemiological profile
Source: Front Microbiol. 2024 Oct 16;15:1462802. doi: 10.3389/fmicb.2024.1462802 (PMC11521903; doi:10.3389/fmicb.2024.1462802)
Supplement: Supplementary file 1 [file Data_Sheet_1.docx]

**Title:** Viral co-detection of influenza virus and other respiratory viruses in hospitalized Brazilian patients during the first three years of the coronavirus disease (COVID)-19 pandemic: An epidemiological profile

**Short title:** Influenza virus and co-detection

**Supplementary Material**

| **Supplementary Table 1.** Places in Brazil where the co-detection was described. | |
| --- | --- |
| **Brazilian states and Federal district** | **N (%)** |
| Amazonas | 16 (3.4%) |
| Bahia | 16 (3.4%) |
| Ceará | 16 (3.4%) |
| Federal District | 27 (5.7%) |
| Espírito Santo | 5 (1.0%) |
| Goiás | 42 (8.8%) |
| Maranhão | 8 (1.7%) |
| Minas Gerais | 32 (6.7%) |
| Mato Grosso do Sul | 8 (1.7%) |
| Mato Grosso | 2 (0.4%) |
| Pará | 7 (1.5%) |
| Paraíba | 3 (0.6%) |
| Pernambuco | 2 (0.4%) |
| Piauí | 3 (0.6%) |
| Paraná | 66 (13.8%) |
| Rio de Janeiro | 19 (4.0%) |
| Rondônia | 2 (0.4%) |
| Roraima | 1 (0.2%) |
| Rio Grande do Sul | 29 (6.1%) |
| Santa Catarina | 16 (3.4%) |
| Sergipe | 1 (0.2%) |
| São Paulo | 156 (32.7%) |

%: percentage, N: number of individuals. The data comprised the period from December 19, 2019, to April 06, 2023 – three years since the beginning of the coronavirus disease (COVID)-19 pandemic in Brazil.

| **Supplementary Table 2.** Distribution of respiratory viruses in hospitalized patients due to severe acute respiratory syndrome caused by influenza virus infection according to seasons. | | | | | |
| --- | --- | --- | --- | --- | --- |
| **Respiratory viruses*** | **Autumn** | **Spring** | **Summer** | **Winter** | **Total** |
|  | **N (%)** | **N (%)** | **N (%)** | **N (%)** |  |
| Influenza A | 74 (18.5) | 98 (24.5) | 179 (44.8) | 49 (12.3) | 400 |
| Influenza B | 14 (18.2) | 12 (15.6) | 45 (58.4) | 6 (7.8) | 77 |
| Adenovirus | 8 (12.5) | 25 (39.1) | 19 (29.7) | 12 (18.8) | 64 |
| Bocavirus | 3 (18.8) | 4 (25.0) | 8 (50.0) | 1 (6.3) | 16 |
| Metapneumovirus | 4 (22.2) | 3 (16.7) | 8 (44.4) | 3 (16.7) | 18 |
| Parainfluenza virus type 1 | 2 (3.9) | 18 (35.3) | 27 (52.9) | 4 (7.8) | 51 |
| Parainfluenza virus type 2 | 2 (11.8) | 5 (29.4) | 7 (41.2) | 3 (17.6) | 17 |
| Parainfluenza virus type 3 | 4 (16.0) | 6 (24.0) | 12 (48.0) | 3 (12.0) | 25 |
| Parainfluenza virus type 4 | 2 (28.6) | 2 (28.6) | 3 (42.9) | 0 (0.0) | 7 |
| Rhinovirus | 10 (14.9) | 22 (32.8) | 24 (35.8) | 11 (16.4) | 67 |
| Respiratory syncytial virus | 64 (25.3) | 36 (14.2) | 130 (51.4) | 23 (9.1) | 253 |
| **Total** | 88 (18.4) | 110 (23.1) | 224 (47.0) | 55 (11.5) | 477 |

%: percentage, N: number of individuals. *: influenza virus infection was associated as the cause of hospitalization, and the other viruses were co-detected with the influenza virus. Autumn from March 21 to June 21. Winter from June 21 to September 23. Spring from September 23 to December 21. Summer from December 21 to March 21. The dates were presented according to the notification periods. The data comprised the period from December 19, 2019, to April 06, 2023 – three years since the beginning of the coronavirus disease (COVID)-19 pandemic in Brazil.

| **Supplementary Table 3.** Distribution of respiratory viruses in hospitalized patients due to severe acute respiratory syndrome caused by influenza virus infection according to the date of notification. * | | | | | | | | | | | |
| --- | --- | --- | --- | --- | --- | --- | --- | --- | --- | --- | --- |
| **Complete date** | **Influenza** | | **Adenovirus** | **Bocavirus** | **Metapneumovirus** | **Parainfluenza virus type** | | | | **Rhinovirus** | **Respiratory syncytial virus** |
|  | **A** | **B** |  |  |  | 1 | 2 | 3 | 4 |  |  |
| 01-Jan-2020 | 2 | 3 | 2 | 0 | 1 | 0 | 1 | 1 | 0 | 0 | 1 |
| 01-Feb-2020 | 5 | 4 | 3 | 0 | 2 | 0 | 0 | 1 | 0 | 1 | 2 |
| 01-Mar-2020 | 21 | 4 | 5 | 0 | 5 | 3 | 1 | 3 | 1 | 7 | 8 |
| 01-Apr-2020 | 2 | 1 | 1 | 0 | 0 | 0 | 1 | 1 | 0 | 0 | 2 |
| 01-May-2020 | 1 | 0 | 0 | 0 | 0 | 0 | 0 | 0 | 0 | 0 | 1 |
| 01-Jun-2020 | 2 | 0 | 0 | 0 | 0 | 0 | 0 | 0 | 0 | 1 | 1 |
| 01-Jul-2020 | 1 | 0 | 0 | 0 | 0 | 0 | 0 | 0 | 0 | 1 | 1 |
| 01-Aug-2020 | 0 | 1 | 0 | 0 | 0 | 0 | 0 | 0 | 0 | 1 | 0 |
| 01-Jan-2021 | 5 | 0 | 0 | 0 | 0 | 4 | 2 | 0 | 0 | 0 | 0 |
| 01-Feb-2021 | 2 | 0 | 0 | 0 | 0 | 0 | 0 | 0 | 0 | 0 | 2 |
| 01-Mar-2021 | 3 | 0 | 0 | 0 | 0 | 1 | 0 | 0 | 0 | 0 | 2 |
| 01-Apr-2021 | 1 | 2 | 0 | 0 | 0 | 0 | 0 | 0 | 0 | 0 | 3 |
| 01-May-2021 | 1 | 0 | 0 | 0 | 0 | 1 | 1 | 0 | 0 | 0 | 0 |
| 01-Jun-2021 | 2 | 1 | 0 | 0 | 0 | 0 | 0 | 0 | 0 | 0 | 3 |
| 01-Jul-2021 | 0 | 1 | 0 | 0 | 0 | 0 | 0 | 0 | 0 | 0 | 1 |
| 01-Aug-2021 | 2 | 1 | 0 | 0 | 0 | 1 | 2 | 0 | 0 | 0 | 1 |
| 01-Sep-2021 | 2 | 0 | 1 | 0 | 0 | 0 | 0 | 0 | 0 | 1 | 0 |
| 01-Oct-2021 | 2 | 1 | 0 | 0 | 0 | 0 | 0 | 1 | 0 | 1 | 1 |
| 01-Nov-2021 | 9 | 3 | 0 | 0 | 0 | 4 | 0 | 2 | 0 | 2 | 6 |
| 01-Dec-2021 | 67 | 4 | 5 | 10 | 1 | 11 | 3 | 7 | 2 | 8 | 32 |
| 01-Jan-2022 | 83 | 9 | 5 | 0 | 0 | 11 | 3 | 5 | 2 | 10 | 59 |
| 01-Feb-2022 | 8 | 1 | 1 | 0 | 0 | 1 | 0 | 0 | 0 | 0 | 7 |
| 01-Mar-2022 | 16 | 3 | 0 | 0 | 0 | 1 | 0 | 0 | 1 | 4 | 14 |
| 01-Apr-2022 | 17 | 3 | 1 | 0 | 1 | 0 | 0 | 0 | 0 | 0 | 18 |
| 01-May-2022 | 17 | 0 | 2 | 2 | 0 | 0 | 0 | 1 | 0 | 0 | 14 |
| 01-Jun-2022 | 27 | 2 | 4 | 1 | 4 | 2 | 0 | 0 | 0 | 5 | 16 |
| 01-Jul-2022 | 15 | 0 | 3 | 0 | 0 | 0 | 1 | 0 | 0 | 4 | 8 |
| 01-Aug-2022 | 6 | 1 | 1 | 0 | 0 | 1 | 0 | 2 | 0 | 1 | 2 |
| 01-Sep-2022 | 18 | 2 | 9 | 1 | 1 | 0 | 0 | 1 | 0 | 2 | 6 |
| 01-Oct-2022 | 31 | 2 | 11 | 0 | 0 | 7 | 2 | 0 | 1 | 11 | 4 |
| 01-Nov-2022 | 15 | 0 | 7 | 0 | 2 | 2 | 0 | 0 | 0 | 1 | 3 |
| 01-Dec-2022 | 4 | 2 | 1 | 0 | 0 | 1 | 0 | 0 | 0 | 1 | 3 |
| 01-Jan-2023 | 4 | 1 | 1 | 0 | 0 | 0 | 0 | 0 | 0 | 0 | 5 |
| 01-Feb-2023 | 3 | 6 | 1 | 2 | 1 | 0 | 0 | 0 | 0 | 2 | 4 |
| 01-Mar-2023 | 6 | 19 | 0 | 0 | 0 | 0 | 0 | 0 | 0 | 3 | 23 |
| **Months** | **A** | **B** | **Adenovirus** | **Bocavirus** | **Metapneumovirus** | **1** | **2** | **3** | **4** | **Rhinovirus** | **Respiratory syncytial virus** |
| January | 94 | 13 | 8 | 0 | 1 | 15 | 6 | 6 | 2 | 10 | 65 |
| February | 18 | 11 | 5 | 2 | 3 | 1 | 0 | 1 | 0 | 3 | 15 |
| March | 46 | 26 | 5 | 0 | 5 | 5 | 1 | 3 | 2 | 14 | 47 |
| April | 20 | 6 | 2 | 0 | 1 | 0 | 1 | 1 | 0 | 0 | 23 |
| May | 19 | 0 | 2 | 2 | 0 | 1 | 1 | 1 | 0 | 0 | 15 |
| June | 31 | 3 | 4 | 0 | 4 | 2 | 0 | 0 | 0 | 6 | 20 |
| July | 16 | 1 | 3 | 1 | 0 | 0 | 1 | 0 | 0 | 5 | 10 |
| August | 8 | 3 | 1 | 0 | 0 | 2 | 2 | 2 | 0 | 2 | 3 |
| September | 28 | 2 | 10 | 1 | 1 | 0 | 0 | 1 | 0 | 3 | 6 |
| October | 33 | 3 | 11 | 0 | 0 | 7 | 2 | 1 | 1 | 12 | 5 |
| November | 28 | 5 | 8 | 0 | 2 | 7 | 0 | 2 | 0 | 4 | 12 |
| December | 67 | 4 | 5 | 10 | 1 | 11 | 3 | 7 | 2 | 8 | 32 |
| **Total** | 400 | 77 | 64 | 16 | 18 | 51 | 17 | 25 | 7 | 67 | 253 |

The numbers represent the number of cases per date or month. *: influenza virus infection was associated as the cause of hospitalization, and the other viruses were co-detected with the influenza virus. The dates were presented according to the notification periods. The data comprised the period from December 19, 2019, to April 06, 2023 – three years since the beginning of the coronavirus disease (COVID)-19 pandemic in Brazil.

| **Supplementary Table 4.** Distribution of respiratory viruses in hospitalized patients due to severe acute respiratory syndrome caused by influenza virus infection according to the date of notification. | | | | | | | | |
| --- | --- | --- | --- | --- | --- | --- | --- | --- |
| **Respiratory viruses*** | **<1 year of age** | **1 to 12 years of age** | **13 to 24 years of age** | **25 to 60 years of age** | **61 to 72 years of age** | **73 to 85 years of age** | **+85 years of age** | **Total** |
|  | **N (%)** | **N (%)** | **N (%)** | **N (%)** | **N (%)** | **N (%)** | **N (%)** |  |
| Influenza A | 117 (29.2) | 141 (35.2) | 15 (3.7) | 59 (14.8) | 15 (3.7) | 34 (8.5) | 19 (4.8) | 400 |
| Influenza B | 27 (35.0) | 29 (37.7) | 1 (1.3) | 9 (11.7) | 3 (3.9) | 7 (9.0) | 1 (1.3) | 77 |
| Adenovirus | 12 (18.7) | 40 (62.5) | 3 (4.7) | 5 (7.8) | 1 (1.6) | 3 (4.7) | 0 (0) | 64 |
| Bocavirus | 3 (18.7) | 11 (68.7) | 1 (6.2) | 0 (0) | 1 (6.2) | 0 (0) | 0 (0) | 16 |
| Metapneumovirus | 4 (22.2) | 6 (33.3) | 1 (5.6) | 5 (27.8) | 0 (0) | 1 (5.5) | 1 (5.5) | 18 |
| Parainfluenza virus type 1 | 2 (3.9) | 11 (21.6) | 5 (9.8) | 13 (25.5) | 4 (7.8) | 11 (21.6) | 5 (9.8) | 51 |
| Parainfluenza virus type 2 | 1 (5.9) | 7 (41.1) | 1 (5.8) | 4 (23.5) | 2 (11.8) | 2 (11.8) | 0 (0) | 17 |
| Parainfluenza virus type 3 | 2 (8.0) | 9 (36.0) | 1 (4.0) | 8 (32.0) | 0 (0) | 4 (16.0) | 1 (4.0) | 25 |
| Parainfluenza virus type 4 | 1 (14.3) | 2 (28.5) | 0 (0) | 3 (42.8) | 1 (14.3) | 0 (0) | 0 (0) | 7 |
| Rhinovirus | 10 (14.9) | 28 (41.8) | 4 (5.9) | 15 (22.4) | 5 (7.5) | 2 (3.0) | 3 (4.4) | 67 |
| Respiratory syncytial virus | 118 (46.6) | 74 (29.2) | 4 (1.6) | 21 (8.3) | 5 (2.0) | 20 (7.9) | 11 (4.3) | 253 |

%: percentage, N: number of individuals. *: influenza virus infection was associated as the cause of hospitalization, and the other viruses were co-detected with the influenza virus. The data comprised the period from December 19, 2019, to April 06, 2023 – three years since the beginning of the coronavirus disease (COVID)-19 pandemic in Brazil.
